# Geophysical evidence of a nearly dry bridgmanite in the Earth’s lower mantle

**DOI:** 10.1038/s41467-026-76621-1

**Published:** 2026-08-12

**Authors:** Yoshiyuki Okuda, Kenji Ohta, Chris E. Mohn, Yu Nishihara, Goru Takaichi, Saori Kawaguchi-Imada, Hirokazu Kadobayashi, Kei Hirose

**Affiliations:** 1 Department of Earth and Planetary Science, The University of Tokyo, Tokyo, Japan; 2 Hawai’i Institute of Geophysics and Planetology, University of Hawai’i at Mānoa, Honolulu, HI USA; 3 Department of Earth and Planetary Sciences, Institute of Science Tokyo, Tokyo, Japan; 4 Centre for Earth Evolution and Dynamics (CEED), University of Oslo, Oslo, Norway; 5 Centre for Planetary Habitability (PHAB), University of Oslo, Oslo, Norway; 6 Department of Chemistry and Center for Materials Science and Nanotechnology, University of Oslo, Oslo, Norway; 7 Geodynamics Research Center, PIAS, Ehime University, Matsuyama, Ehime Japan; 8 SPring-8, Japan Synchrotron Radiation Research Institute, Hyogo, Japan; 9 Earth-Life Science Institute, Tokyo Institute of Technology, Tokyo, Japan

**Keywords:** Geophysics, Solid Earth sciences

## Abstract

Water in Earth’s mantle plays a crucial role in shaping geological and geochemical processes. While previous electrical conductivity measurements on major mantle minerals have constrained water content in the Earth’s mantle down to the mantle transition zone, the water content of the lower mantle remains experimentally unconstrained. This study measures the electrical conductivity of hydrous (Fe,Al)-bearing bridgmanite, synthesized with Fe and Al compositions representative of peridotitic lithology, at pressures up to 47 GPa and temperatures up to 2210 K. The results demonstrate that proton conduction is the predominant charge transport mechanism in hydrous (Fe,Al)-bearing bridgmanite under the investigated pressure-temperature conditions. Water incorporation markedly enhances the electrical conductivity of bridgmanite, increasing it by approximately an order of magnitude relative to that of dry samples. Extrapolation of the experimental data suggests that bridgmanite in the lower mantle contains less than 40–70 wt. ppm H_2_O near the top and at mid-mantle depths. These findings offer insight into potential hidden water reservoirs in Earth’s lower mantle.

## Abstract (web summary)

Electrical conductivity measurements indicate that bridgmanite in the present-day lower mantle contains little water, providing constraints on water storage and transport in Earth’s deep interior.

## Introduction

The amount and distribution of water within the Earth’s interior directly influence key physical and chemical properties, thereby shaping the planet’s thermochemical evolution. A substantial portion of Earth’s water is thought to be incorporated as impurities within nominally anhydrous silicate minerals in the crust and mantle^1^. The electrical conductivity (EC) of minerals is highly sensitive to their water content. Laboratory measurements of EC on upper mantle and transition zone minerals—such as olivine^2,3^, pyrope garnet^4^, orthopyroxene^5^, clinopyroxene and plagioclase^6,7^, wadsleyite and ringwoodite^8–10^—as well as subducted slab materials^11,12^, combined with electromagnetic observations, have provided critical constraints on the water content of the Earth’s upper mantle and transition zone.

As the Earth’s largest layer, accounting for more than half of the planet’s total volume, even a small amount of water in the lower mantle can significantly impact the silicate Earth’s overall water budget^1^. The pyrolitic lower mantle is thought to consist of (Fe,Al)-bearing bridgmanite, ferropericlase, and davemaoite, with bridgmanite being the dominant mineral, comprising approximately 80% of the assemblage^13^. As such, bridgmanite is considered to primarily control the EC of the Earth’s lower mantle^14^. Although retrogressed former bridgmanite has been identified in natural inclusions within superdeep diamonds^15^, no direct analyses of water content in natural bridgmanite have been reported to date. This absence underscores the need for laboratory-based EC measurements to constrain the water abundance in lower mantle bridgmanite. Numerous studies have measured the EC of bridgmanite using multi-anvil presses^14,16–20^ and diamond anvil cells (DAC)^21–29^ across a range of Fe and Al contents. However, the EC of hydrous bridgmanite has not yet been experimentally investigated, and current understanding relies largely on theoretical calculations^30,31^. Estimates of water in the Earth’s lower mantle have been inferred from the water solubility of (Fe,Al)-bearing bridgmanite, which provides an upper bound on water content. Reported values vary widely, ranging from less than 5 to approximately 4000 wt. ppm H_2_O, likely due to differences in chemical composition^32–38^. Nonetheless, experimental studies using bridgmanite samples with compositions close to peridotite consistently yield water contents exceeding several hundred wt. ppm H_2_O^33,34,36,38^.

Here we report high-pressure (*P*) and temperature (*T*) EC measurements of hydrous (Fe,Al)-bearing bridgmanite, with a composition resembling that of the peridotitic lower mantle, at 34–47 GPa and up to 2210 K. The measured conductivity is approximately an order of magnitude higher than that of dry (Fe,Al)-bearing bridgmanite^28^. Combining our results with geomagnetic observations, we estimate that bridgmanite in the lower mantle is nearly dry, containing less than 40–70 wt. ppm H_2_O near the top of the lower mantle. This estimate is significantly lower than previous geochemical estimates of mantle water content (~200–300 wt. ppm H_2_O^39,40^), and places important constraints on the Earth’s global water budget and deep water cycle.

## Results

### High *P-T* EC measurements

**Fig. 1:**
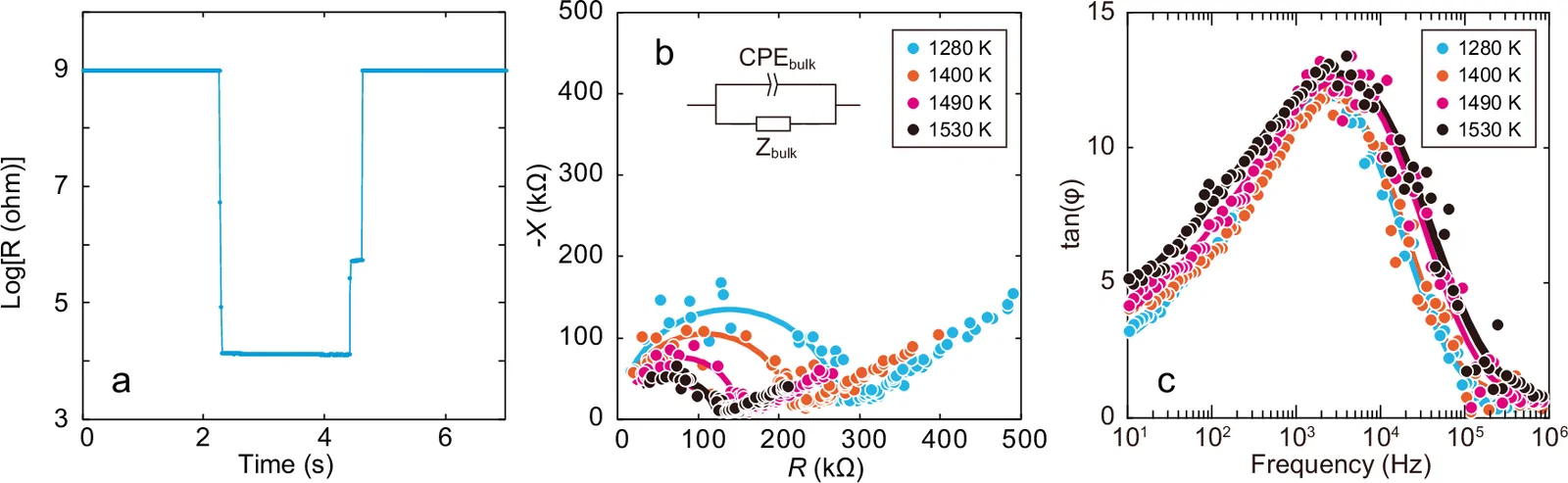
Electrical resistance and impedance data for hydrous bridgmanite sample. **a** Time evolution of resistance during heating to 2020 K at 47 GPa in run #2 for the 293 wt. ppm H_2_O bridgmanite sample. Resistance of **~**10^9^ Ω observed before and after temperature quench is the detection limit of the Sourcemeter used in this measurement. **b** Impedance spectra of hydrous bridgmanite collected from 10^1^ to 10^6^ Hz at high temperature in run #3 for the 177 wt. ppm H_2_O bridgmanite sample. Colored curves show the fitting results using Eq. (7). **c** Dielectric loss tangent tanφ as a function of frequency. Colored curves show the fitting results using Eq. (12).

The EC of hydrous (Fe,Al)-bearing bridgmanite resembling pyrolitic composition with two different water contents was measured in a laser-heated DAC. We synthesized two hydrous bridgmanite samples using a 3000-ton Kawai-type MA apparatus (ORANGE-3000) at Ehime University, following a similar manner described in the previous study^41^. The water contents in the samples were estimated to be 293 ± 18 and 177 ± 26 ppm in weight by Fourier Transform Infrared Spectroscopy (FTIR) analysis (see “Methods”). Four and two independent runs were conducted for 177 and 293 wt. ppm H_2_O-bearing bridgmanite samples, respectively (Table 1). We employed a recently developed method for measuring the EC of materials that are transparent to near-infrared lasers used for heating in a DAC^42^ (see “Methods”). The direct current electrical resistance of the sample was measured at high *P-T* (Fig. 1a), while we also collected impedance spectra in run #3 for the 177 wt. ppm H_2_O bridgmanite at high *P-T*, and run #2 for the 293 wt. ppm H_2_O bridgmanite sample at room temperature following the high-temperature experiment (Fig. 1b and Supplementary Fig. 1).

**Table 1:** Experimental conditions and electrical conductivity of hydrous bridgmanite

H_2_O (wt. ppm)	Run #	Pressure (GPa)	Measured *T* (K)	Simulated sample *T* (K)	Sample thickness (µm)	Laser spot size (µm)	log [*R* (Ohm)]	log [*σ* (S/m)]	log [*D* (m^2^/s)]
177 (26)	1	34 (2)	1690 (170)	1590 (160)	27.8 (6)	23.0 (23)	4.92 (1)	−0.09 (9)	−7.86 (17)
		35 (2)	2010 (200)	1890 (190)			4.58 (1)	0.25 (9)	−7.45 (17)
		35 (2)	1960 (200)	1850 (180)			4.61 (1)	0.21 (9)	−7.49 (17)
		35 (2)	1890 (190)	1780 (180)			4.67 (1)	0.15 (9)	−7.57 (17)
		35 (2)	1810 (180)	1710 (170)			4.77 (1)	0.05 (9)	−7.68 (17)
		35 (2)	1740 (170)	1630 (160)			4.88 (1)	−0.0 5 (9)	−7.81 (17)
		34 (2)	1660 (170)	1560 (160)			4.94 (1)	−0.12 (9)	−7.89 (17)
		34 (2)	1580 (160)	1490 (150)			5.04 (1)	−0.22 (9)	−8.01 (17)
		34 (2)	1510 (150)	1420 (140)			5.18 (1)	−0.35 (9)	−8.17 (17)
177 (26)	2	37 (2)	1600 (160)	1500 (150)	28.7 (8)	23.0 (23)	5.14 (1)	−0.30 (9)	−8.09 (17)
		37 (2)	1670 (170)	1570 (160)			5.06 (1)	−0.22 (9)	−7.99 (17)
		37 (2)	1810 (180)	1700 (170)			4.88 (1)	−0.04 (9)	−7.78 (17)
		37 (2)	1950 (190)	1830 (180)			4.72 (1)	0.12 (9)	−7.59 (17)
		38 (2)	2090 (210)	1960 (200)			4.60 (1)	0.23 (9)	−7.44 (17)
		38 (2)	2010 (200)	1890 (190)			4.65 (1)	0.18 (9)	−7.51 (17)
		37 (2)	1880 (190)	1760 (180)			4.80 (1)	0.04 (9)	−7.68 (17)
		37 (2)	1740 (170)	1640 (160)			4.93 (1)	−0.09 (9)	−7.85 (17)
		37 (2)	1610 (160)	1510 (150)			5.11 (1)	−0.27 (9)	−8.06 (17)
		37 (2)	1470 (150)	1380 (140)			5.32 (1)	−0.48 (9)	−8.31 (17)
177 (26)	3	37 (2)	1370 (140)	1280 (130)	17.5 (8)	17.3 (17)	5.52 (5)	−0.65 (9)	−8.51 (17)
		37 (2)	1500 (150)	1400 (140)			5.31 (5)	−0.43 (9)	−8.26 (17)
		38 (2)	1640 (160)	1530 (150)			5.06 (5)	−0.19 (9)	−7.97 (17)
		37 (2)	1590 (160)	1490 (150)			5.16 (5)	−0.28 (9)	−8.08 (17)
		37 (2)	1530 (150)	1430 (140)			5.24 (5)	−0.37 (9)	−8.18 (17)
		37 (2)	1420 (140)	1320 (130)			5.43 (5)	−0.56 (9)	−8.40 (17)
177 (26)	4	43 (2)	1970 (200)	1890 (190)	25.9 (17)	30.0 (30)	4.47 (1)	0.10 (11)	−7.60 (18)
		43 (2)	1800 (180)	1730 (170)			4.66 (1)	−0.10 (10)	−7.83 (18)
		42 (2)	1480 (150)	1420 (140)			5.15 (2)	−0.58 (11)	−8.40 (18)
		43 (2)	1590 (160)	1520 (150)			4.97 (2)	−0.40 (11)	−8.19 (18)
		43 (2)	1630 (160)	1560 (160)			4.88 (1)	−0.32 (11)	−8.09 (18)
		43 (2)	1780 (180)	1710 (170)			4.67 (1)	−0.11 (11)	−7.85 (18)
		43 (2)	1940 (190)	1860 (190)			4.52 (1)	0.05 (11)	−7.65 (18)
		44 (2)	2090 (210)	2010 (200)			4.38 (1)	0.18 (11)	−7.48 (18)
293 (18)	1	35 (2)	1970 (200)	1880 (190)	24.5 (11)	22.7 (23)	4.28 (4)	0.50 (11)	−7.41 (16)
		35 (2)	1830 (180)	1750 (170)			4.45 (3)	0.34 (10)	−7.61 (16)
		35 (1)	1700 (170)	1620 (160)			4.60 (3)	0.18 (10)	−7.80 (16)
		35 (1)	1560 (160)	1480 (150)			4.77 (3)	0.01 10)	−8.01 (16)
		34 (2)	1420 (140)	1350 (140)			5.00 (3)	−0.22 (10)	−8.27 (16)
293 (18)	2	47 (5)	2230 (220)	2210 (220)	10.2 (4)	21.3 (21)	4.00 (4)	0.45 (11)	−7.39(16)
		47 (5)	2110 (210)	2090 (210)			4.06 (3)	0.40 (11)	−7.47(16)
		47 (5)	1970 (200)	1950 (200)			4.14 (2)	0.32 (10)	−7.58 (16)
		46 (5)	1840 (180)	1820 (180)			4.24 (2)	0.21 (10)	−7.71 (15)
		46 (5)	1700 (170)	1690 (170)			4.34 (2)	0.11 (10)	−7.85 (15)
		46 (5)	1570 (160)	1560 (160)			4.45 (2)	0.00 (10)	−8.00 (15)
		46 (5)	1770 (180)	1750 (180)			4.30 (2)	0.16 (10)	−7.79 (15)
		47(5)	2040 (200)	2020 (200)			4.11 (3)	0.34 (10)	−7.54 (16)
		46 (5)	1910 (190)	1890 (190)			4.21 (2)	0.25 (10)	−7.66 (15)
		46 (5)	1770 (180)	1750 (180)			4.31 (2)	0.15 (10)	−7.80 (15)
		46 (5)	1640 (160)	1620 (160)			4.41 (2)	0.05 (10)	−7.93 (15)
		46 (5)	1500 (150)	1490 (150)			4.52 (2)	−0.07 (10)	−8.08 (15)
		45 (5)	1370 (140)	1360 (140)			4.67 (1)	−0.22 (10)	−8.27 (15)
		43 (4)	300	300			9.29 (1)	−6.17 (10)	−14.9 (2)

**Fig. 2:**
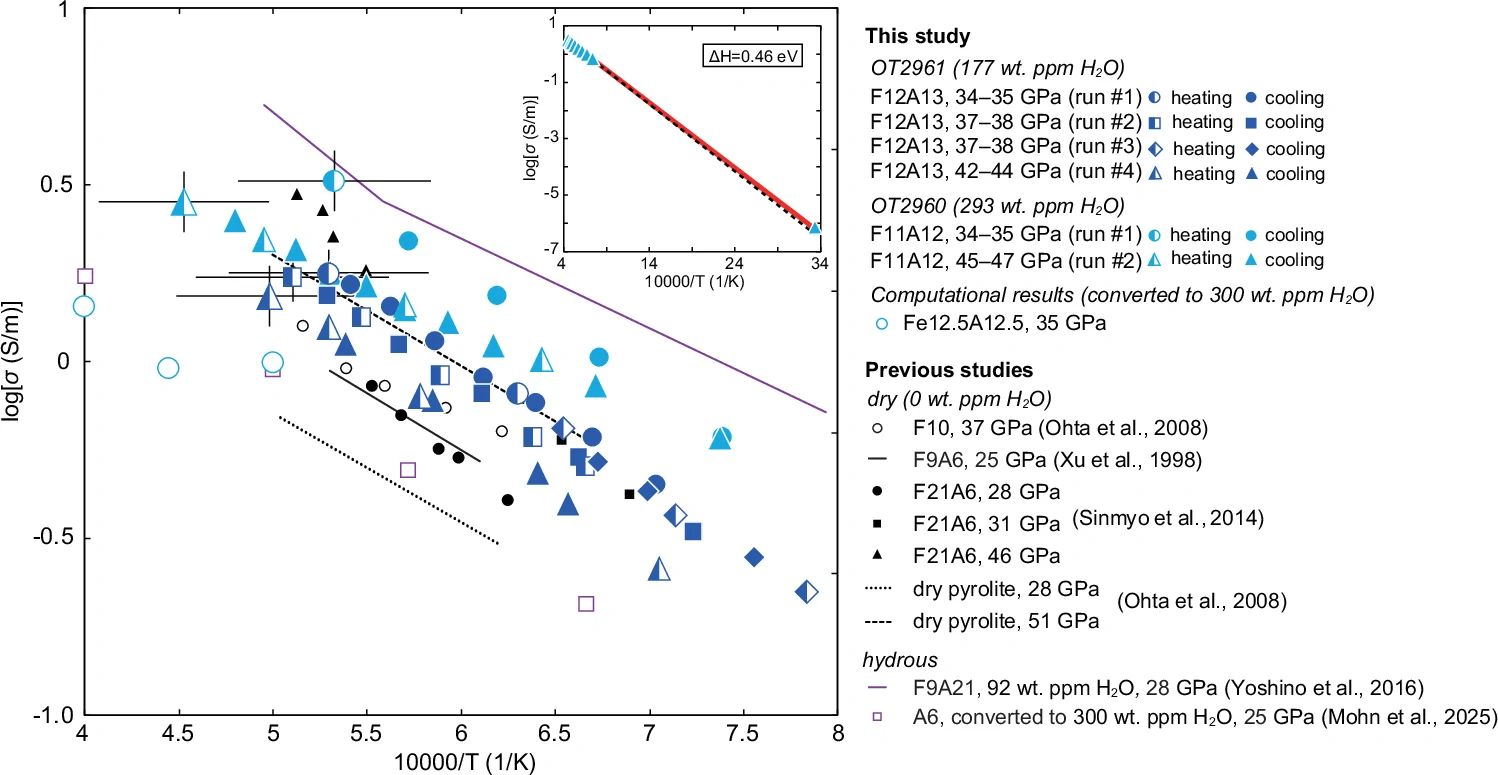
Electrical conductivity of hydrous bridgmanite. Blue symbols, 177 wt. ppm H_2_O (Fe,Al)-bearing bridgmanite at 34–35 GPa in run #1 (circle), 37–38 GPa in run #2 (square), 37–38 GPa in run #3 (diamond), and 42–44 GPa in run #4 (triangle). Light blue symbols, 293 wt. ppm H_2_O (Fe,Al)-bearing bridgmanite at 34–35 GPa in run #1 (circle), and 45–47 GPa in run #2 (triangle). Half-filled and closed symbols represent data collected during heating and cooling, respectively. Error bars are shown only for the highest *P–T* datum in each run for clarity. Light blue open circles represent the calculated proton conductivity of (Fe,Al)-bearing bridgmanite at 35 GPa carried out using ab initio MD simulations (this study). The water content in the calculation was 1000 ppm H_2_O, where the water content was converted to 300 ppm when calculating the electrical conductivity using Eq. (3). The slight deviation from linearity in the calculated results is attributed to numerical errors. Open black circles represent dry Fe 10 mol%-bearing bridgmanite^22^. Filled symbols represent dry (Fe,Al)-bearing bridgmanite conductivity data on Fe 9 mol%, Al 6 mol% bridgmanite at 25 GPa (solid line)^19^, Fe 21 mol%, Al 6 mol% bridgmanite at 28 GPa (circle), 31 GPa (square), and 46 GPa (triangle)^28^. Dotted and broken lines indicate dry pyrolite conductivity data^23^. Purple solid line indicates (Fe,Al)-bearing hydrous bridgmanite data on Fe 9 mol%, Al 21 mol% bridgmanite with 92 wt. ppm H_2_O^20^. The inset figure is the electrical conductivity of 293 wt. ppm H_2_O bridgmanite obtained in run #2 with room temperature datum plotted together. The solid red line represents the regression line for conductivity data, including room temperature data point, while the black dashed line shows the regression line for high-temperature data extrapolated to room temperature.

The EC of hydrous bridgmanite at 34–47 GPa is summarized in Fig. 2. In run #1 for the bridgmanite sample containing 177 wt. ppm H_2_O, the sample was initially compressed to 34 GPa at room temperature. It was then heated from 1590 to 1890 K, followed by a cooling cycle to 1420 K, while collecting sample resistance. During the heating cycle, the common logarithm of EC (S/m) increased from −0.09 at 1590 K to 0.25 at 1890 K and subsequently decreased to −0.35 at 1420 K during cooling. The data exhibited a single linear trend in the Arrhenius plot (logarithm of EC vs. reciprocal temperature), indicating a consistent conduction mechanism across the temperature range. Additionally, the agreement between the heating and cooling cycle data suggests that the sample did not experience dehydration, which could otherwise decrease the conductivity due to a reduction in primary charge carriers (see the next section for details). In run #2, conducted at a similar pressure of 37–38 GPa, the logarithm of EC (S/m) increased from −0.30 at 1500 K to 0.23 at 1960 K during heating and decreased to −0.48 at 1380 K during cooling. These results closely aligned with those of run #1, demonstrating the reproducibility of the measurements. High *P–T* impedance measurements conducted in run #3 at comparable pressures of 37–38 GPa yielded consistent results, confirming the reliability of the conductivity values obtained from direct current measurements. The impedance spectra are characterized by a semicircular arc corresponding to the bulk response of the sample, followed by a low-frequency straight-line feature in the Cole–Cole representation (Fig. 1b), consistent with ionic migration and interfacial polarization at the sample–electrode interface. Further details are provided in the Discussion section. In run #4, conducted at a higher pressure of 43–44 GPa, the logarithm of EC (S/m) also followed a linear trend in the Arrhenius plot. However, the conductivity values were approximately 20–40% lower than those observed in runs #1 and #2 at equivalent temperatures, indicating a negative pressure dependence of conductivity in hydrous bridgmanite (Fig. 2).

Two experimental runs were conducted with the higher water concentration sample (293 wt. ppm H_2_O). In run #1, performed at pressures comparable to those in runs #1 and #2 for the 177 wt. ppm H₂O-bearing samples (34–35 GPa), the sample was heated from approximately 1350 to 1880 K. The logarithm of EC (S/m) increased from −0.22 to 0.50, following a single linear trend in the Arrhenius plot. Consistency between the heating and cooling cycle data suggests that dehydration did not occur, even with the higher water content. The conductivity of the sample containing 293 wt. ppm H_2_O was approximately 1.7 times greater than that containing 177 wt. ppm H_2_O at 35–37 GPa. Based on the Nernst-Einstein equation, proton conductivity is proportional to water concentration (i.e., number of protons). The observed 1.7-fold difference in conductivity aligns well with the difference in water concentration between the two hydrous bridgmanite samples, indicating that protons were the primary charge carriers in the experiment. Run #2 was conducted at a higher pressure of 46–47 GPa and showed lower conductivity values compared to run #1. This indicates a negative pressure dependence of EC in the 293 wt. ppm H_2_O-bearing bridgmanite sample, consistent with the trend observed in the 177 wt. ppm H_2_O-bearing one. Impedance spectroscopy was performed after the high-temperature experiment in this run. The sample resistance at room temperature was determined to be 1.93(9) GΩ (see “Methods”), yielding a logarithm EC (S/m) of −6.17(10). This value agrees with the conductivity extrapolated to room temperature based on high-temperature data (Fig. 2), indicating that the conduction mechanism remained consistent across room and high temperatures. Our experimental results were generally consistent with our computational results for hydrous pyrolitic (Fe,Al)-bearing bridgmanite at the similar pressure, as well as with previous calculations on Al-bearing bridgmanite^30^; the difference in EC from our ab initio calculations and experimental data was approximately twofold at 1500 K, increasing to 4.3 times at 2000 K due to the weaker temperature dependence in the computational results compared to the experimental data (Fig. 2).

## Discussion

### High proton conductivity in hydrous (Fe,Al)-bearing bridgmanite

Previous studies have proposed that the primary charge carrier in dry (Fe,Al)-bearing bridgmanite is the small polaron, which exhibits a positive pressure dependence on EC^28^. In contrast, our data show that hydrous bridgmanite shows a negative pressure dependence, with EC proportional to the water content in the sample. Proton conduction is widely recognized as the dominant charge transport mechanism at relatively low temperatures, primarily due to its low activation energy, which is a consequence of the small size and low mass of protons enabling rapid diffusion^43^. The consistency between the room temperature EC data extrapolated from high-temperature data and the directly measured room temperature EC (Fig. 2) supports that proton conduction is the primary mechanism in hydrous (Fe,Al)-bearing bridgmanite containing 177 and 293 wt. ppm H_2_O within the examined *P-T* range of 33–47 GPa and 300–2210 K. Furthermore, the proton diffusion coefficients derived from a detailed analysis of the impedance data are consistent with those estimated from EC values, providing additional evidence that protons are the primary charge carriers (see the following section for details). The persistence of proton conduction as the dominant transport mechanism at high temperatures contrasts with the behavior of other mantle minerals, such as olivine and wadsleyite, in which small polaron conduction becomes dominant above ~1000 and ~1700 K, respectively^3^. The sustained high proton conductivity observed in bridgmanite at high temperatures suggests significantly higher proton mobility in bridgmanite than in other minerals.

The collected EC data for hydrous (Fe,Al)-bearing bridgmanite were fitted using the Arrhenius equation:

1 $$\sigma={\sigma }_{0}\exp \left(\frac{-\Delta H}{{kT}}\right)$$

where *σ*_0_, Δ*H*, *k*, and *T* are the pre-exponential factor, activation enthalpy, Boltzmann constant, and absolute temperature, respectively. Supplementary Table 1 summarizes the fitting results for each experimental run, with Δ*H* and log*σ*_0_ as fitting parameters. The obtained Δ*H* values ranged from 0.47 to 0.75 eV, depending on water content and *P-T* conditions. These values are consistent with those reported for proton conduction in other mantle minerals, such as olivine, wadsleyite, and ringwoodite, which typically exhibit Δ*H* < 1 eV^2,3,8,9,43^. To further examine the water dependence, we also fitted the EC data obtained at identical pressures of 34–35 GPa for bridgmanite containing 177 and 293 wt. ppm H_2_O, using a modified Arrhenius equation that incorporates the effect of water concentration on both the pre-exponential factor and the activation enthalpy^43^:

2 $$\sigma={\sigma }_{0p}{C}_{w}\exp \left(\frac{-{H}_{p}^{0}-\alpha {C}_{w}^{\frac{1}{3}}}{{kT}}\right)$$

where *σ*_0p_ is pre-exponential factor, *C*_w_ is water concentration in weight percent, *H*_p_^0^ is activation enthalpy observed at zero hydrogen concentrations, *α* is a constant accounting for geometrical factors showing the dependence of the proton conduction activation enthalpy on water content. The fitting results yielded log[*σ*_0p_ (S/m/wt%)] = 3.83(3), *H*_p_^0^ = 0.730(18) eV, and *α* = 0.12(5). Previous studies reported that the water-concentration dependence of activation enthalpy is negligible in wadsleyite (*α* = 0.02), whereas ringwoodite exhibits a stronger dependence (*α* = 0.62)^9^. Hydrous bridgmanite showed a slightly higher *α* value than wadsleyite, although it remains relatively small. A small *α* value indicates that proton conduction in hydrous bridgmanite follows the Nernst-Einstein relation, implying that proton transport is governed by normal proton hopping^9^.

The measured proton conductivity in bridgmanite exhibits a negative pressure dependence, in agreement with previous theoretical predictions^30,31^. This observation implies that an alternative conduction mechanism—most likely small polarons—may become the dominant charge carrier at higher pressures. Consequently, proton conduction in bridgmanite is likely to be significant only in the shallower regions of the Earth’s lower mantle. Although theoretical studies of Al-H-bearing bridgmanite predict a reversal of this trend at higher pressures, resulting in a positive pressure dependence^30^, our experiments did not extend to such conditions, and thus we cannot access this possibility further.

The proton conductivity of the 293 wt. ppm H_2_O bridgmanite measured in this study was five times higher than the previously reported small polaron conductivity in (Fe,Al)-bearing bridgmanite at similar pressures of 31 GPa^28^ and 35 GPa (this study). It was also approximately an order of magnitude higher than the EC of pyrolite at 28 GPa^23^, which is considered representative of pyrolitic bridgmanite^14^, and higher than other reported values for dry Fe- and (Fe,Al)-bearing bridgmanite at 23–24 GPa^14,17–19,22^. These results collectively demonstrate high proton conductivity in (Fe,Al)-bearing bridgmanite. In contrast, both bridgmanite samples measured in this study showed lower conductivity than (Fe,Al)-bearing bridgmanite containing 93 wt. ppm H_2_O at 28 GPa^20^ (Fig. 2). Yoshino et al. ^20^. reported that small polaron conduction was the most likely conduction mechanism, although they acknowledged that proton conduction could not be ruled out. The high EC observed in their study may partly reflect the negative pressure dependence of proton conductivity in hydrous bridgmanite, indicating that enhanced proton conduction could have contributed to the elevated EC in their measurement. In addition, their sample composition (~9 mol% Fe and ~21 mol% Al) differs from that of our samples (~11–12 mol% Fe and ~12–13 mol% Al), which may also have contributed to the discrepancy between the two studies.

Fe-bearing bridgmanite without Al has been reported to exhibit reduced conductivity due to the spin transition of Fe^22,24^. While the occurrence of a spin transition of Fe in (Fe,Al)-bearing bridgmanite remains debated, theoretical calculations suggest that such a transition is unlikely to occur in (Fe,Al)-bearing bridgmanite^44,45^. Furthermore, experimental evidence indicates that Fe spin transitions in (Fe,Al)-bearing bridgmanite would require synthesis pressures significantly higher than those used in this study^46,47^. Therefore, the observed negative pressure dependence of EC in our samples is more plausibly explained by the suppression of proton hopping mobility at higher pressures, rather than by a spin transition.

### Proton diffusion coefficient of bridgmanite

**Fig. 3:**
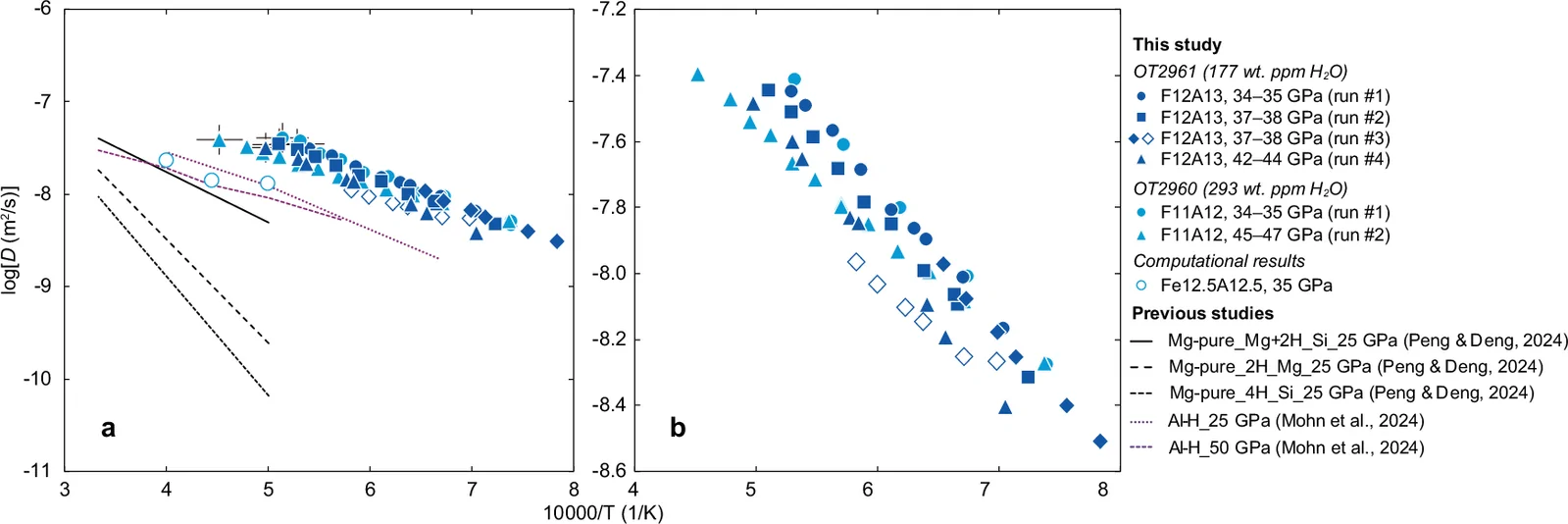
Proton diffusion coefficient in bridgmanite. **a** Proton diffusion coefficients obtained in this study compared with previous theoretical calculations. **b** Expanded view showing only the data from this study. Blue symbols represent 177 wt. ppm H_2_O (Fe,Al)-bearing bridgmanite at 34–35 GPa in run #1 (circle), 37–38 GPa in run #2 (square), 37–38 GPa in run #3 (diamond), and 42–44 GPa in run #4 (triangle). Light blue symbols represent 293 wt. ppm H_2_O (Fe,Al)-bearing bridgmanite at 34–35 GPa in run #1 (circle), and 45–47 GPa in run #2 (triangle). Blue open diamonds show the proton diffusion estimated from impedance data using Eq. (4). Error bars are shown only for the highest *P–T* datum in each run for clarity. Light blue open circles represent the calculated proton diffusion coefficient of (Fe,Al)-bearing bridgmanite at 35 GPa carried out using ab initio MD simulations (this study). The black solid, dotted and broken lines denote the theoretically calculated proton diffusion coefficients for Mg-pure hydrous bridgmanite with substitution mechanisms of Mg^2+^ + 2H^+^↔Si^4+^, 2H^+^↔Mg^2+^, and 4H^+^↔Si^4+^, respectively^31^. Purple dotted and broken lines denote the theoretically calculated proton diffusion coefficients for Al-bearing hydrous bridgmanite with the substitution mechanism of Al^3+^ + H^+^↔Si^4+^ at 25 and 50 GPa, respectively^30^.

Proton conductivity is influenced by the concentration of mobile charge carriers and is therefore proportional to the water content of the sample. In contrast, the proton diffusion coefficient (*D*_*p*_) reflects the intrinsic mobility of protons, independent of their concentration. *D*_*p*_ can be estimated from proton conductivity (*σ*_*p*_) using the Nernst-Einstein relation:

3 $${\sigma }_{p}=\frac{n{z}^{2}{{{\rm{F}}}}^{2}{D}_{p}}{{{\rm{R}}}T}$$

where *R* is gas constant, *T* is absolute temperature, *n* is the number density of protons in mol/m^3^, *z* is valency (proton = 1), and *F* is Faraday’s constant. Figure 3 presents the *D*_*p*_ of bridgmanite estimated from measured EC using Eq. (3). Notably, the *D*_*p*_ values of bridgmanite containing 293 wt. ppm H_2_O at 34–35 GPa are nearly identical to those of the 177 wt. ppm H_2_O sample, suggesting similar proton mobility in these samples. This observation is consistent with previous computational results indicating that water concentration has a negligible effect on *D*_*p*_ in hydrous bridgmanite^31^.

The *D*_*p*_ of bridgmanite is sensitive to composition and can vary by 1–2 orders of magnitude depending on the available hydrogen incorporation pathways^31^. The similarity in *D*_*p*_ for the two samples at identical pressures suggests that proton site occupancies were similar. The temperature dependence of *D*_*p*_ determined in this study aligns well with MD simulations for Al-bearing^30^ and Al-free^31^ bridgmanite, with the latter involving the substitution of Mg^2+^ and 2H^+^ for Si^4+^, as well as with theoretical calculations presented in this study (Supplementary Table 2). Experimentally obtained *D*_*p*_ values showed reasonable agreement with the calculated ones, being 2.16 times higher at 1500 K and 4.15 times higher at 2000 K. The observed difference may reflect compositional effects. The dominant hydrogen substitution mechanism in (Fe,Al)-bearing bridgmanite is likely a Tschermak-type substitution, in which Al^3+^ and H^+^ replace Si^4+^ at the B site^48^. Peng and Deng^31^ demonstrated that protons incorporated via Tschermak-like substitution, where Mg^2+^ and 2H^+^ replace Si^4+^, exhibit the highest *D*_*p*_ in Al-free bridgmanite. These values are consistent with those for Al-bearing bridgmanite assuming a Tschermak-type substitution^30^. However, recent neutron diffraction experiments suggest that this mechanism alone cannot fully explain the observed proton site occupancies, particularly configurations involving O–H–O bonds between octahedral oxygen sites, which remain to be fully identified^38^. Recent combined first-principles and spectroscopic studies further indicate that hydrogen in bridgmanite can be incorporated through multiple defect mechanisms, including Mg and Si vacancies that accommodate a significant fraction of the total water content, as well as coupled substitutions such as Al^3+^ + H^+^ that increase the diversity of proton sites^49^. Given that *D*_*p*_ is strongly dependent on proton incorporation mechanisms^31^, the presence of such additional hydrogen configurations may contribute to the relatively high proton conductivity observed in this study.

We also independently estimated the *D*_*p*_ through a detailed analysis of the impedance spectra. *D*_*p*_ was calculated using the following equation^50^:

4 $${D}_{p}=\frac{{d}^{2}}{\tau {\delta }^{2}}$$

where *d* is half the distance between two electrodes (2*d* = sample thickness), and *τ* and *δ* are the dielectric relaxation time and a dimensionless parameter, respectively. Here, *τ* corresponds to the characteristic time scale at which electrode polarization, originating from double-layer formation at the electrode interface, becomes dominant. The parameters *τ* and *δ* were obtained by fitting the loss tangent (tan *ϕ*)^50^, which in our setup is expressed as the ratio *R/X* (Fig. 1c; see “Methods” for details). At room-temperature, *D*_*p*_ for the 293 wt. ppm H_2_O-bearing bridgmanite sample in run #2 is 2.9(14) × 10^−3^ µm^2^/s, in close agreement with the value independently derived from EC data using Eq. (3), 2.6(11) × 10^−3^ µm^2^/s. High *P–T D*_*p*_ for the 177 wt. ppm H_2_O-bearing bridgmanite sample in run #3 also agrees with the values derived from EC data within uncertainty (Fig. 3). The close correspondence between the two estimates supports the robustness of the *D*_*p*_ values obtained in this study.

One might argue that grain boundary diffusion could contribute to the elevated conductivity and diffusion coefficients observed in experimental studies, including the present work, as the EC of mantle minerals can be significantly influenced by grain boundary diffusion^51^. Accounting for the grain boundary diffusion, the effective diffusion coefficient (*D*_eff_) can be expressed as follows^52^:

5 $${D}_{{{\rm{eff}}}}={D}_{{{\rm{bulk}}}}+\frac{3{\delta }_{g}}{{d}_{g}}{D}_{{{\rm{gb}}}}$$

where *D*_bulk_ is bulk diffusion coefficient, *δ*_*g*_ is grain boundary width, *d*_*g*_ is grain size (10–30 μm in the present experiments), and *D*_gb_ is grain boundary diffusion coefficient, respectively. To the best of our knowledge, no data have been reported for the proton grain boundary diffusion coefficient in bridgmanite. However, assuming that it is comparable to that of other mantle minerals^53^, and adopting a typical *δ*_*g*_ value of 1 nm^54^, the estimated contribution of grain boundary diffusion ($\frac{3{\delta }_{g}}{{d}_{g}}{D}_{{{\rm{gb}}}}$) is approximately 1–2 orders of magnitude smaller than the measured diffusion coefficients. Moreover, EC dominated by grain boundary water typically exhibits a much smaller activation enthalpy, i.e., nearly constant conductivity in an Arrhenius plot^55^, which was not observed in this study. These suggest that grain boundary diffusion did not significantly contribute to the measured conductivity.

### EC of hydrous bridgmanite in the Earth’s lower mantle

**Fig. 4:**
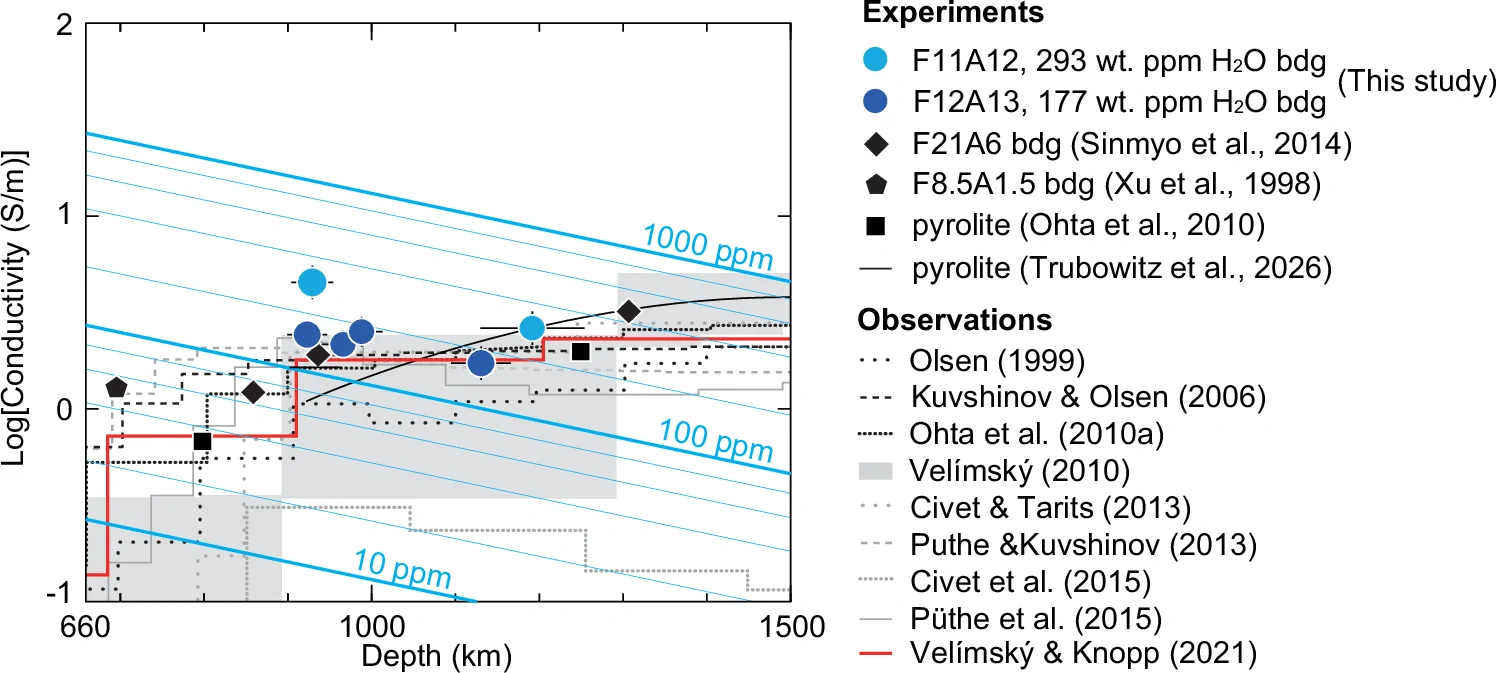
One-dimensional depth profiles of the electrical conductivity of hydrous bridgmanite in the Earth’s lower mantle. Light blue and dark blue symbols represent the electrical conductivity of 293 and 177 wt. ppm H_2_O-bearing bridgmanite (bdg). Blue lines represent the estimated proton conductivity of bridgmanite at 10, 100, and 1000 wt. ppm H_2_O, with additional minor lines at intermediate values evenly spaced on the logarithmic scale. Error bars on conductivity represent uncertainties propagated from experimental measurements. Depth uncertainties were derived from pressure uncertainty. Black square, diamond, and pentagon represent the previous laboratory measurements of pyrolite^23^, (Fe,Al)-bearing bridgmanite^28^, and Fe 8.5, Al 1.5 mol% bridgmanite^19^, respectively. Black solid curve represents the previous laboratory-based conductivity profile for pyrolite^56^. Black dotted, broken, and dense dotted lines, and gray band, dotted, broken, dense dotted, and solid lines represent the profiles obtained by the Occam inversion from the observed C-response data from Olsen^60^, Kuvshinov & Olsen^59^, Ohta et al. ^23^, Velímský^63^, Civet & Tarits^58^, Püthe & Kuvshinov^62^, Civet et al. ^57^, and Püthe et al. ^61^, respectively. Red line represents the most recent observation data (CUP20-OSC5 model) from Velímský & Knopp^64^.

The ECs of dry pyrolite and Fe,Al-bearing bridgmanite^23,28,56^ are slightly higher than, but generally consistent with, the one-dimensional average mantle EC depth profiles derived from electromagnetic induction studies, which range between studies from ~0.1 to 1 S/m at depths of 660–1000 km^23,57–64^. To assess the average water content in the Earth’s lower mantle, we calculated the one-dimensional EC depth profile for bridgmanite containing 177 and 293 wt. ppm H_2_O using Eq. (1), the fitted *σ*_0_ and Δ*H* values, and a representative geotherm^65^. At depths below 1000 km, the ECs of hydrous bridgmanite with both 177 and 293 wt. ppm H_2_O exceeded those of dry pyrolite and Fe,Al-bearing bridgmanite^23,28^. Notably, bridgmanite containing 293 wt. ppm H_2_O exhibits EC values approximately 3–10 times higher than the average mantle conductivity at these depths (Fig. 4). These results suggest that a lower mantle composed of bridgmanite with 177–293 wt. ppm H_2_O cannot explain the observed EC depth profile, implying that the average water content in the upper to mid-lower mantle must be lower than these values. Although Ohta et al. ^23^, Sinmyo et al. ^28^, and this study adopt different geotherms, their influence on the results is minor (Supplementary Fig. 2). Depth EC profiles of hydrous bridgmanite as a function of water content were estimated by extrapolating our hydrous bridgmanite EC data, assuming a linear dependence on water concentration^43^ (Fig. 4). The effect of oxygen fugacity was not considered in these calculations^66^. Our modeling indicates that an average water content of ~5–20 wt. ppm H_2_O in bridgmanite at the top of the lower mantle is most consistent with electromagnetic observations^23,57–64^. Comparison with recent satellite-based electromagnetic sounding results derived from Swarm and CryoSat-2^64^ suggests ~5 wt. ppm H_2_O at 660–700 km and ~30–40 wt. ppm H_2_O at 700–900 km depth. At these depths, we found that, below ~40–70 wt. ppm H_2_O, small-polaron conductivity of dry Fe,Al-bearing bridgmanite^19^ or dry pyrolite^23^ exceeds proton conductivity estimated from hydrous bridgmanite (Supplementary Fig. 3). This crossover indicates that, below this water content, the bulk conductivity of bridgmanite is expected to be dominated by small-polaron conduction rather than proton conduction, making it difficult to resolve lower water contents from conductivity alone. We therefore conclude that bridgmanite in these depth ranges contains <40–70 wt. ppm H_2_O. This estimate assumes that the Fe-Al content of lower-mantle bridgmanite is comparable to that of our experimental samples and to that expected for bridgmanite in a pyrolitic lower-mantle assemblage^67,68^. As discussed above, the comparison with Yoshino et al. suggests that proton conductivity in bridgmanite may also be sensitive to Fe-Al composition. If lower-mantle bridgmanite has substantially different Fe-Al contents or Fe valence states^69^, the inferred water-content threshold may change accordingly.

The inferred water contents are substantially lower than geochemical estimates, which suggest ~200–300 wt. ppm H_2_O in the mantle^39,40^. At depths greater than ~900 km, the EC of hydrous bridgmanite even with both 177 and 293 wt. ppm H_2_O converges with that of dry pyrolite and Fe,Al-bearing bridgmanite^23,28^ (Fig. 4). This suggests that small polaron conduction dominates at these depths, thereby making it difficult to estimate water content in the deeper lower mantle. Assuming a vertically uniform water content of <40–70 wt. ppm H_2_O throughout the lower mantle, the total amount of water stored in bridgmanite would correspond to less than 0.07 times the mass of Earth’s oceans. This is significantly lower than previous estimates of up to 2.9 ocean masses^1^. While such a homogeneous water distribution is plausible given the fully convective nature of the lower mantle, localized hydrous regions may exist, for example, a deep primordial water reservoir, as inferred from the anomalously low D/H ratios in Ocean Island Basalts^70,71^.

Given that the Earth’s building blocks may contain a large amount of water^72^, bridgmanite crystallizing from a magma ocean is expected to incorporate water up to its solubility limit. Previous studies indicate that water preferentially partitions into the melt phase^73^, while a significant fraction can remain in the solid, implying that lower mantle bridgmanite may constitute one of the largest water reservoirs following magma ocean crystallization^74^. However, the estimated water content in bridgmanite at the upper portion of the lower mantle (660–900 km), <40–70 wt. ppm H_2_O, is significantly lower than recent estimates of water solubility in pyrolitic bridgmanite, which is above several hundred wt. ppm H_2_O^33,34,36,38^. The low water contents inferred in this study suggest that bridgmanite in the lower mantle has lost a substantial fraction of its water at some point between its formation and the present day. Proton diffusion is considered to play a negligible role in water loss in the Earth’s lower mantle^30,31^, implying that alternative mechanisms must be responsible for the observed depletion.

Due to the disproportionation of ferrous iron in Fe,Al-bearing bridgmanite, ~1 wt.% metallic iron is thought to form in the lower mantle, where it is believed to be trapped in grain boundaries of lower mantle minerals^75^. Hydrogen is known to be highly siderophile at high *P-T* conditions^76,77^. Indeed, previous experimental studies have shown that H_2_O strongly partitions into metallic iron over mantle silicates such as olivine, wadsleyite, and ringwoodite^78,79^. Although the partitioning behavior of H_2_O between metallic iron and bridgmanite has not yet been experimentally quantified, a significant amount of hydrogen may preferentially partition into metallic iron at grain boundaries. This introduces the possibility that water in the Earth’s lower mantle is primarily stored in metallic iron, and that the overall water content of the Earth’s lower mantle could be higher than previously thought, despite the present study geophysically indicating that lower mantle bridgmanite itself is dry. Note that such iron hydrides, trapped isolated in grain boundaries, would not contribute to the bulk lower mantle EC. Given the relatively low melting temperature of iron hydride, it might have percolated down to the core rather than remained stored in grain boundaries (see Supplementary Discussion for further discussion). The geophysical evidence presented in this study, demonstrating that lower mantle bridgmanite is dry, places critical constraints on the water content and deep water cycle in Earth’s lower mantle, and the potential for hidden hydrogen reservoirs in Earth’s interior.

## Methods

### Sample synthesis

The starting materials were oxide mixtures of talc (Mg_3_Si_4_O_10_(OH)_2_), brucite (Mg(OH)_2_), fayalite (Fe_2_SiO_4_), MgO, and Al_2_O_3_, which were packed into an Au capsule, and then loaded into a MgO container. Two synthesis experiments were conducted (runs OT2960 and OT2961) by compressing the starting material to 25 GPa and heating it for 12 h at 1700 and 1500 °C, respectively. We used a LaCrO_3_ heater for heating, and the synthesis temperature was monitored with a W3Re–W25Re thermocouple. The recovered samples both consisted of two phases: quenched hydrous melt and bridgmanite. The bridgmanite crystals had sizes of ~50–200 µm and was identified by XRD measurement using a Rigaku RAPID II-V/DW with Cu Kα1 radiation (Supplementary Fig. 4). The chemical compositions of bridgmanite were determined to be Mg_0.842(4)_Fe_0.114(3)_Al_0.122(2)_Si_0.922(4)_O_3_ for 293 wt. ppm H_2_O-bearing sample and Mg_0.817(6)_Fe_0.118(5)_Al_0.125(3)_Si_0.940(7)_O_3_ for 177 wt. ppm H_2_O-bearing sample, by Energy Dispersive Spectroscopy analysis using Thermoscientific Helios 5 UC at the University of Tokyo.

FTIR spectra were collected using a JASCO IRT-5200EUO at Ehime University under vacuum conditions with a BaF_2_ window. Single grains of bridgmanite samples synthesized in runs OT2960 and OT2961 were double-side polished and cleaned multiple times, first with acetone and then with ethanol, to remove potential contaminants. We collected unpolarized FTIR spectra from three and two different locations for OT2960 and OT2961 bridgmanite samples, respectively, with 128 scans per location over the range of 600–7000 cm^−1^ using an aperture of 50 × 50 µm^2^. Water content was determined using the equation^80^:

6 $${C}_{{OH}}=\frac{{X}_{i}}{150\xi }\int \frac{K(\nu )}{(3780-\nu )}d\nu$$

where *C*_OH_ is the concentration of hydroxyl in ppm wt. H_2_O, *ξ* is an orientation factor of 1/3 for unpolarized spectra, *K*(*ν*) is the absorption coefficient in cm^−1^ for a given wavenumber *ν*, and *X*_*i*_ is a density factor calculated as *X*_*i*_ = 10^6^ × (18/2*d*), in which *d* is mineral density, respectively. We used the *X*_*i*_ of bridgmanite with pyrolitic composition estimated in a previous study of 2123 ppm wt. H_2_O^34^. The FTIR spectra were corrected by subtracting a linear baseline from 3000 to 3780 cm^−1^. The average water content by weight was calculated as 293 ± 18 ppm for OT2960 and 177 ± 26 ppm for OT2961 hydrous bridgmanite samples. These values are lower than those reported for hydrous (Fe,Al)-bearing bridgmanite^33^, likely due to differences in iron and aluminum content (Supplementary Fig. 5), as well as a potential systematic underestimation of water content by the Paterson calibration, as recent work suggests that the absorption coefficient of bridgmanite deviates from the Paterson calibration^49^. While a recent FTIR study suggested near-zero water capacity in (Fe,Al)-bearing bridgmanite^35^, a neutron diffraction study showed ~1000 wt. ppm H_2_O can be incorporated into (Fe,Al)-bearing bridgmanite, providing strong evidence for the incorporation of a significant number of structural protons^38^. Although not detected in our XRD measurements, a minor brucite phase was observed in the FTIR spectra (Supplementary Fig. 5a), as previously reported^32,34^. The brucite peak was excluded when estimating the water content of the sample. The inclusion of a minor phase does not significantly affect EC measurements as long as it is not interconnected in a sample; the EC of brucite at high *P-T* conditions has been reported to be lower than that of bridgmanite^81^, and thus isolated brucite inclusions may slightly underestimate the bulk conductivity. Brucite was not detected in the XRD patterns, indicating that, if present, its abundance is at most a few vol.%. Based on effective medium bounds for isolated inclusions^82^, such a small volume fraction would result in an underestimation of up to only a few percent, suggesting a minor effect on the measured conductivity. However, brucite may decompose into MgO + H_2_O under the *P-T* conditions of this study^83^, which could cause an irreversible jump in conductivity due to highly conductive H_2_O fluid in grain boundaries^84^. Nonetheless, no such conductivity jump was observed in this study (see “Results” for details). Recent studies also indicate that the water solubility of bridgmanite increases with temperature^74^, raising the possibility that water released from brucite could be incorporated into bridgmanite, gradually modifying its water content and, in turn, its EC. However, the measured conductivity shows a linear dependence on temperature, suggesting that such rehydration effects did not significantly influence the results.

### EC measurement in LH-DAC

EC measurements were conducted using a laser-heated DAC with diamonds having 300 µm culets. A hole matching the diameter of the anvil culet was drilled at the center of a pre-indented rhenium gasket with a thickness of ~30 µm. The hole was filled with a mixture of cubic boron nitride (cBN) and TiO_2_ (binder), which was then compressed to a thickness of ~45–50 µm. A 50 µm diameter sample chamber was created by drilling a hole in the cBN layer using a UV laser. A single-crystal bridgmanite sample with ~50 µm in diameter was polished to ~15–30 µm in thickness, then loaded into the sample chamber, sandwiched between a boron-doped diamond (BDD) layer (3 µm thick and ~50 µm wide), acting as both a laser absorber and resistance probe, and 10–15 µm-thick ZrO_2_ thermal insulation layers (Supplementary Fig. 6); in run #3 for 177 wt. ppm H_2_O-bearing bridgmanite, iridium electrodes were placed in direct contact with the sample. The BDD or iridium was connected to platinum foils on both sides of the gasket, which were further connected to a copper wire, a lead wire, and a source meter or an impedance analyzer. The entire DAC assembly after the sample was loaded was placed in a dry desiccator for more than one day, and the sample chamber was sealed right after taken out.

We conducted two and four independent runs for 293 and 177 wt. ppm H_2_O-bearing hydrous bridgmanite samples, respectively. All the runs were conducted at the BL10XU beamline of the SPring-8 synchrotron radiation facility^85^, except run #2 for 293 wt. ppm H_2_O-bearing bridgmanite, which was performed at UTokyo. We employed a pair of 100 W single-mode Yb fiber lasers (BL10XU, SPI Lasers Co. Ltd.; UTokyo, IPG Photonics) with beam-shaping optics enabling flat-top intensity distribution to heat the hydrous bridgmanite sample via BDD electrodes from both sides. We did not observe any chemical reactions between electrodes and bridgmanite samples (Supplementary Fig. 7). The sample was heated by applying a given laser power for 2 s, followed by quenching. The heating duration was deliberately limited to 2 s to minimize any potential sample dehydration. These heating cycles were repeated at varying temperatures by increasing the laser output power in each run. Throughout the process, we simultaneously recorded the electrical resistance and thermal radiation spectra from the sample, together with X-ray diffraction patterns for experiments performed at SPring-8 (Supplementary Fig. 8). The laser-heated spot was approximately 30 μm across, much larger than the size (approximately 6 μm in diameter) of a focused incident X-ray beam. The temperature of the electrode surface during heating was determined with an EMCCD camera detecting system (ProEM, Teledyne Princeton Instruments) coupled with a spectrometer. The collected thermal radiation spectra from the sample were fitted by Planck’s grey body radiation law to calculate temperatures. The temperature of the hydrous bridgmanite samples between the two electrodes is essentially lower than such measured temperature; it was calculated by a three-dimensional finite element method using a software package COMSOL Multiphysics (COMSOL Inc.) (Supplementary Fig. 9). Temperature distributions were calculated for each experiment based on the measured laser spot size and sample thickness. A homogeneous circular area, equivalent to the diameter of the laser spot, was considered on both sample surfaces, with heat propagation occurring through the sample, the surrounding 10 µm-thick zirconia layers, and the diamond anvils. The back surface of the diamond anvils was set at room temperature. The thermal conductivity of the sample was taken from the previously reported thermal conductivity of (Fe,Al)-bearing bridgmanite^86^. The thermal conductivity of zirconia was set at 10 W/m/K, which is a typical value for corundum under high *P-T* conditions^87^. Tetrahedral meshes with a resolution of 0.1 µm were applied. We performed simulations for each run and estimated a three-dimensional average temperature in the region of the cylindrical dimension with the top having laser spot size (= experimental sample temperature). The difference between the temperature measured at the electrode surface and the simulated sample temperature was 1–5% (Table 1). The temperature uncertainty was assumed to be ±10%. Experimental pressure except run #2 for 293 wt. ppm H_2_O-bearing sample was determined using the unit cell volume of ZrO_2_ based on its thermal equation of state (EoS)^88^. In run #2 for 293 wt. ppm H_2_O-bearing sample, the pressure was obtained by Raman spectroscopy measurements of a diamond anvil^89^, and the effect of thermal pressure was added, employing the empirical relation of 5% increase per 1000 K^90^.

The electrical resistance of the bridgmanite samples was obtained using a pseudo-four-terminal method with a SourceMeter (Keithley 2450), applying a constant 1 mA source current with an uncertainty of 0.012%. Resistance data were collected 4000 times per heating cycle over a 2-s interval. To eliminate the potential influence of the Seebeck voltage, which may arise from temperature heterogeneity within the sample, the direction of the applied current was reversed alternately during measurements (Supplementary Fig. 10). The resistance showed a significant drop upon heating and remained nearly constant until the sample was quenched (Fig. 1a). The sample resistance was estimated by averaging the resistance data collected during heating. The uncertainty in high-temperature resistance was calculated from the standard deviation of the measured resistance values, typically ~1%. In run #2 for the 293 wt. ppm H₂O-bearing bridgmanite sample, we collected an impedance spectrum at room temperature following the high-temperature experiment using a Frequency Response Analyzer FRA51615 (NF Block Corp.). The alternating voltage was set to 0.1 V, and the frequency range was 10^1^–10^4^ Hz. The impedance spectrum was collected in approximately a few minutes. In run #3, for the 177 wt. ppm H_2_O-bearing bridgmanite sample, a HIOKI 3532-80 chemical impedance analyzer was used, with an AC voltage amplitude of 0.1 V and a frequency range of 10^1^–10^6^ Hz. The acquisition of a single spectrum required approximately 30 s. The impedance *Z*(*f*) = *R*(*f*) + *iX*(*f*), where *R*(*f*) and *X*(*f*) are real and imaginary terms, respectively, is presented as a Cole–Cole plot (Fig. 1b). We obtained the sample resistance by fitting a parallel circuit model comprising resistance *R* and a constant phase element (CPE) to each spectrum using the commercial software Zview (Scribner Associates Inc.). The impedance of *R*-CPE parallel circuit is given by:

7 $$Z=\frac{R}{1+{RC}{\left(2\pi {if}\right)}^{p}}$$

where *C*, *f*, *i*, and *p* are capacitance, frequency, imaginary unit, and a fitting parameter (*p* = 1 corresponds to an ideal capacitor), respectively.

The EC of the hydrous bridgmanite sample at high *P-T* was determined from the following equation:

8 $$\sigma \left(P,T\right)=\frac{l}{{RS}}$$

where *R* is the measured resistance at high *P-T*, and *l* and *S* are sample thickness and surface area, respectively. The sample thickness was measured after the sample recovery from a DAC. Its cross-section was sectioned using a focused Ga ion beam (FEI, Versa 3D DualBeam). The effect of elastic deformation on the sample thickness was corrected using the EoS of MgSiO_3_ bridgmanite^91^. We did not consider the effect of volume expansion upon heating, but it is at most a few % only, which is much smaller than a change in the sample resistance (>100%) with increasing temperature. The *S* value at high temperatures was considered identical to the laser spot size, assuming that the electric current induced by an applied electric field flows only in a laser-heated portion. This is a reasonable assumption since EC of minerals increase by several orders of magnitude when elevating temperatures^42^. For room temperature data, *S* was estimated from the overlapped area between the sample and the electrodes. The overall uncertainty in EC, *u*_*EC*_, was calculated as;

9 $${u}_{{EC}}=\sqrt{{u}_{L}^{2}+{u}_{S}^{2}+{u}_{R}^{2}}$$

in which *u*_*L*_ is the standard deviation of the observed sample thickness (~10%), *u*_*S*_ is the uncertainty in the surface area (~10%), and *u*_*R*_ is from the fitting error (a few %). We found *u*_*EC*_ to be typically ~25% (Table 1).

### Estimation of diffusion coefficient from impedance spectra

Unlike electrons, protons cannot pass through the BDD electrodes employed in this study, known as blocking electrodes. When such blocking electrodes are used, protons begin to migrate at the electrode surface at low frequencies, forming a layer of opposite charges at the electrode-sample interface. This phenomenon, referred to as an electrical double layer, introduces an additional low frequency component that is commonly modeled as a constant-phase element and appears as a straight-line feature in the impedance response^92^. The collected impedance spectra indeed showed not only a semi-circular response but also an additional straight-line feature in the low-frequency region. The real (*ε*_1_) and imaginary (*ε*_2_) parts of the dielectric function can be calculated from the obtained complex impedance data as follows:

10 $${\varepsilon }_{1}=\frac{-2{dX}}{\omega {\varepsilon }_{0}S\left({R}^{2}+{X}^{2}\right)}$$

11 $${\varepsilon }_{2}=\frac{2{dR}}{\omega {\varepsilon }_{0}S\left({R}^{2}+{X}^{2}\right)}$$

where 2*d* is the sample thickness, *ε*_0_ is the permittivity in free space, and *S* is the electrode surface area. The loss tangent (tan*ϕ*) of the sample, which is the ratio of *ε*_2_ to *ε*_1_, can be written as follows:

12 $${\tan} {\phi }=\frac{{\varepsilon }_{2}}{{\varepsilon }_{1}}=\frac{\omega \tau \sqrt{\delta }}{1+{\left(\omega \tau \right)}^{2\left(1-a\right)}}$$

where *τ* is the dielectric relaxation time, *δ* is a dimensionless ratio of sample thickness to Debye length, and *a* is a constant (*a* < 1)^50,93^. The diffusion coefficient obtained from impedance analysis using Eq. (4) represents an effective diffusivity of mobile charge carriers under blocking-electrode conditions. Assuming proton-dominated conduction, this effective diffusivity can be interpreted in terms of proton transport. The proton diffusion coefficient was then estimated from the fitted *τ* and *δ* values using Eq. (4), after normalization to the independently determined proton concentration of each sample.

### Computational methods

Calculations of *D* and EC in (Fe,Al) bearing bridgmanite are also carried out using ab initio molecular dynamics simulations together with a jump diffusion model^94–97^:

13 $$D=\frac{1}{6}\sum {f}_{i}{\varGamma }_{i}{a}_{i}^{2}$$

Here, *Γ* and *a*_*i*_ are the jump frequency and the jump distance, respectively. The index *i* refers to the jump directions, which may be between nearest-neighbor resident cavities aligned in the crystallographic <100> direction, next-nearest neighbors aligned in the <110> direction, third-nearest neighbors aligned in the <111> direction, etc. Correlation factors, *f*_*i*_, which measure the correlations between successive jumps of a proton, can be calculated using *f*_*i*_ = 1 + 2<cos(*θ*_*l*,*l*+1_)>, where *θ*_*l*,*l*+1_ is the angle between two consecutive jumps *l* and *l* + *1*. Inspection of the trajectories shows that the proton jumps take place rapidly between mainly nearest neighboring residential proton positions, with little evidence for a correlation between successive jumps. Indeed, we find that *f* is between 0.9 and 1.0 depending on *T*, consistent with a previous MD study on iron-free bridgmanite^30^. From *D*, we can calculate the proton conductivity using Eq. (3).

Investigation of different plausible mechanisms for incorporating protons in iron-free bridgmanite shows that the exchange of Si^4+^ with Al^3+^ + H^+^ is the energetically most favorable mechanism to incorporate protons in the crystal structure at lower mantle conditions. Moreover, computational and experimental studies (including this work) show that both ferric and ferrous iron are high-spin and preferentially located at the crystallographic *A-*site in bridgmanite (whereas Al occupies the *B-*site in lower mantle peridotite^45^).

With these constraints on composition and occupancy of minor elements in bridgmanite in lower mantle peridotite, proton diffusivity is calculated by inserting a single proton within an 81-simulation cell with composition: (Mg_0.875_Fe^2+^_0.0625_Fe^3+^_0.0625_)(Al_0.125_Si_0.875_)O_3_ (Supplementary Fig. 11). This composition and crystal structure are very similar to those investigated experimentally in this work, which allows for a direct comparison between the proton conductivity calculated from theory and experiment. The supercell was constructed by doubling the conventional orthorhombic perovskite-structured MgSiO_3_ along the two crystallographic *a* and *b* directions. Within this supercell, there are many different ways of distributing iron and aluminum on the A and B positions, respectively. Previous computational studies show that Fe^3+^ at the A-site and Al^3+^ at the B-site prefer to align themselves as nearest neighbors even at lower mantle temperature to minimize local lattice strain, whereas Fe^2+^ and Mg^2+^ are distributed at random over the A positions^45^. In this work, we therefore calculate proton diffusion in a configuration where Fe^3+^ and Al^3+^ at the B-site are aligned as nearest neighbors in the simulation box.

The MD simulations ran in the *NVT* ensemble at *T* = 2000, 2250, and 2500 K, with cell volume, *V*, tuned at each temperature to match an average total pressure of 35 GPa. The calculated average pressure after equilibration in the MD runs was 35.2 ± 1.5 GPa at 2000 K, 35.1 ± 1.6 GPa at 2250 K, and 35.3 ± 1.8 GPa at *T* = 2500 K. We use a Nosé–Hoover thermostat, and the step lengths in the MD runs were 0.5 or 1 *fs*. Between 90 and 175 proton jumps were counted in each run. All MD simulations were carried out using VASP^98–100^ with the Perdew-Burke-Ernzerhof functional^100^, an energy cut-off fixed at 400 eV for the electronic wave function and the gamma point only for sampling the Brillouin zone. The electronic entropy is included using Fermi-Dirac statistics with a width k_B_*T* to ensure correct electron-ion coupling at high temperature^101,102^.

To investigate the possible influence on self-interaction errors associated with iron’s 3*d* electrons, we also carry out a molecular dynamics calculation at 3000 K and 35 GPa using an on-site Hubbard *+U* term of 4 eV, which is a reasonable average value used for studying iron-bearing bridgmanite^45,103^. Although the dynamics is hampered by frequent difficulty in converging the wave function at particular configurations we were able to record 27 jumps in 19 ps by restarting the runs using either new forces drawn from a Maxwell–Boltzmann distribution and/or restarting from initial positions using a constant small step length of 0.5 fs in the MD runs without too large drift/changes in the total energy (<25 meV/ps/atom). The calculated diffusion coefficient and average pressure from this run was 2.7 × 10^−8^ m^2^/s at 35.5 ± 2.1 GPa, in very good agreement with that calculated without any on-site Hubbard term in iron (*D* = 3.1 × 10^−8^ m^2^/s) at 34.6 ± 2.1 GPa.

## Supplementary information

Supplementary information

Transparent Peer Review file

## Data Availability

The data supporting the main findings of this study are available in the paper and its Supplementary Information.
