# Supplementary material for: Geophysical evidence of a nearly dry bridgmanite in the Earth’s lower mantle
Source: Nat Commun. 2026 Aug 12;17:9684. doi: 10.1038/s41467-026-76621-1 (PMC13558727; doi:10.1038/s41467-026-76621-1)
Supplement: Supplementary file 1 [file 41467_2026_76621_MOESM1_ESM.pdf]

Supplementary information

Geophysical evidence of a nearly dry bridgmanite in the Earth's lower mantle

Yoshiyuki Okuda[1,2*], Kenji Ohta[3], Chris E. Mohn[4,5,6], Yu Nishihara[7], Goru Takaichi[7], Saori Kawaguchi-Imada[8], Hirokazu Kadobayashi[8] & Kei Hirose[1,9]

[1]Department of Earth and Planetary Science, The University of Tokyo, 7-3-1 Hongo, Bunkyo-ku, Tokyo 113-0033, Japan

[2]Hawai'i Institute of Geophysics and Planetology, University of Hawai'i at Mānoa, Honolulu, Hawaii 96822, USA

[3]Department of Earth and Planetary Sciences, Institute of Science Tokyo, Meguro, Tokyo 152-8551, Japan

[4]Centre for Earth Evolution and Dynamics (CEED), University of Oslo, N-0315 Oslo, Norway

[5]Centre for Planetary Habitability (PHAB), University of Oslo, N-0315 Oslo, Norway

[6]Department of Chemistry and Center for Materials Science and Nanotechnology, University of Oslo, Oslo 0371, Norway

[7]Geodynamics Research Center, PIAS, Ehime University, Matsuyama, Ehime 790-8577, Japan

[8]SPring-8, Japan Synchrotron Radiation Research Institute, Sayo, Hyogo 679-5198, Japan

[9]Earth-Life Science Institute, Tokyo Institute of Technology, Meguro, Tokyo 152-8551, Japan

Supplementary Figures

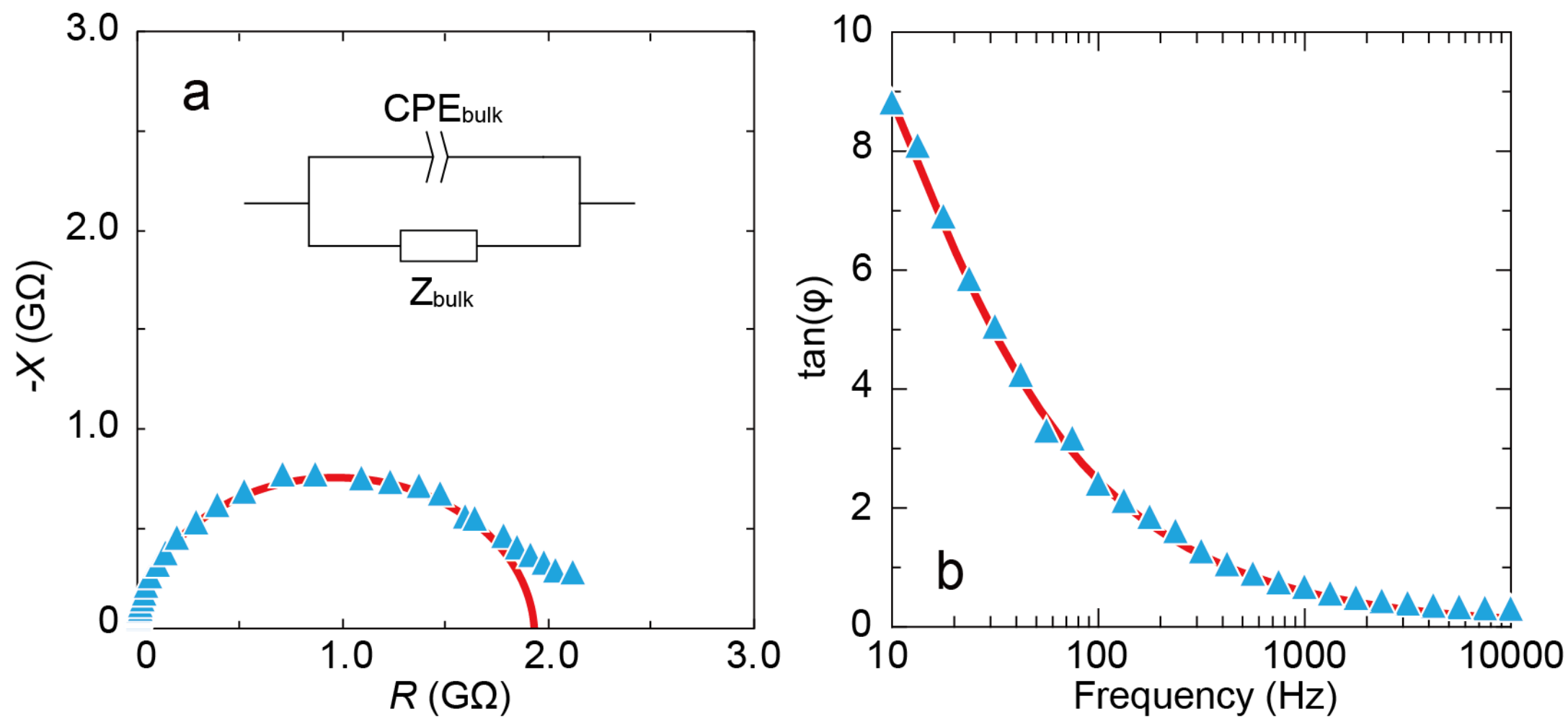

Supplementary Fig. 1 | Impedance data for hydrous bridgmanite sample in run #2 for the 293 wt. ppm H_2O bridgmanite sample. a, Impedance spectra of hydrous bridgmanite collected from 10^1 to 10^4 Hz at room temperature in run #2. Red curve shows the fitting results using Eq. (7). **b,** Dielectric loss tangent tanφ as a function of frequency. Red curve shows the fitting results using Eq. (12).

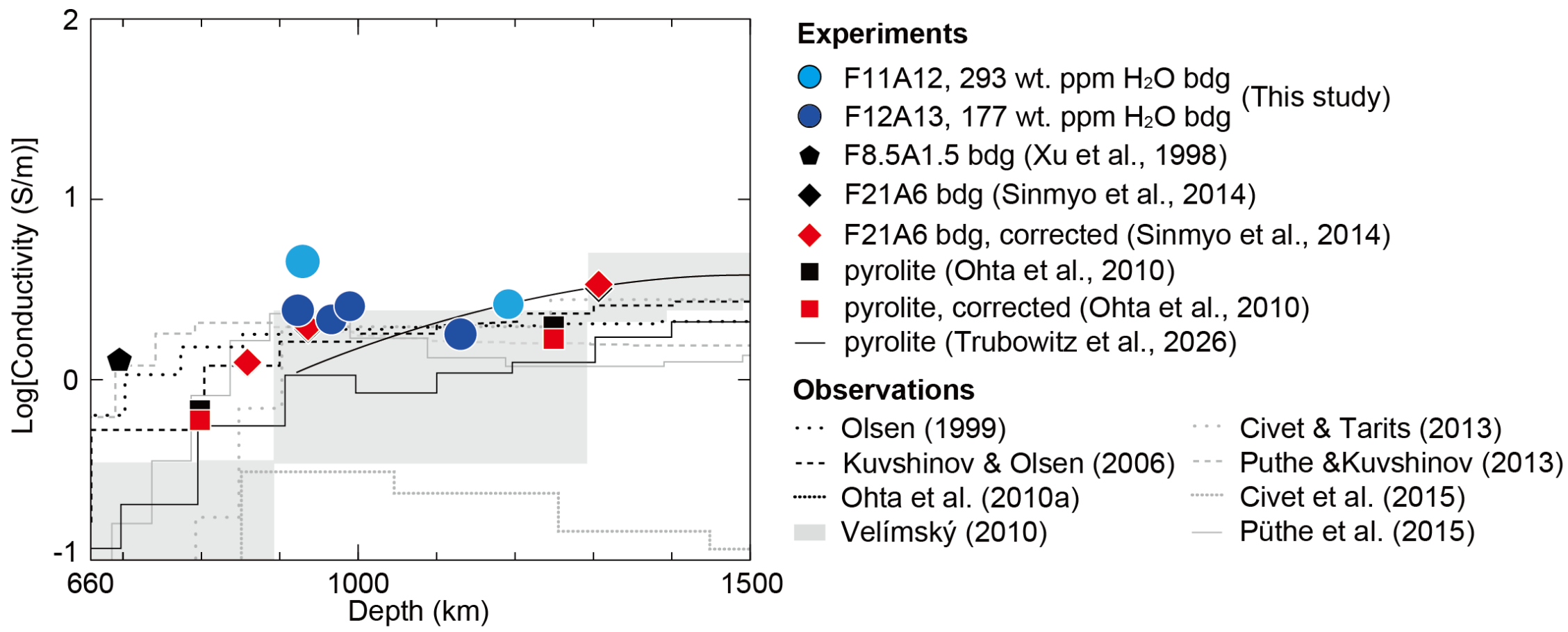

Supplementary Fig. 2 | Electrical conductivity of hydrous bridgmanite plotted together with previous pyrolite and dry Fe,Al-bearing bridgmanite results using the same geotherm (Katsura[1]). Light blue and dark blue symbols represent the electrical conductivity of 293 and 177 wt. ppm H_2O-bearing bridgmanite (bdg). Black square, diamond, and pentagon represent the previous laboratory measurements of pyrolite[2], (Fe,Al)-bearing bridgmanite[3], and Fe 8.5, Al 1.5 mol% bridgmanite[4], respectively. Red square and diamond represent the conductivity of pyrolite[2] and (Fe,Al)-bearing bridgmanite[3], corrected using the geotherm used in this study[1]. Black solid curve represents the previous laboratory-based conductivity profile for pyrolite[5] using Katsura et al.[1]'s geotherm. Black dotted, broken, and dense dotted lines, and gray band, dotted, broken, dense dotted, and solid lines represent the profiles obtained by the Occam inversion from the observed C-response data from Olsen[6], Kuvshinov & Olsen[7], Ohta et al.[2], Velímský[8], Civet & Tarits[9], Püthe & Kuvshinov[10], Civet et al.[11], and Püthe et al.[12], respectively.

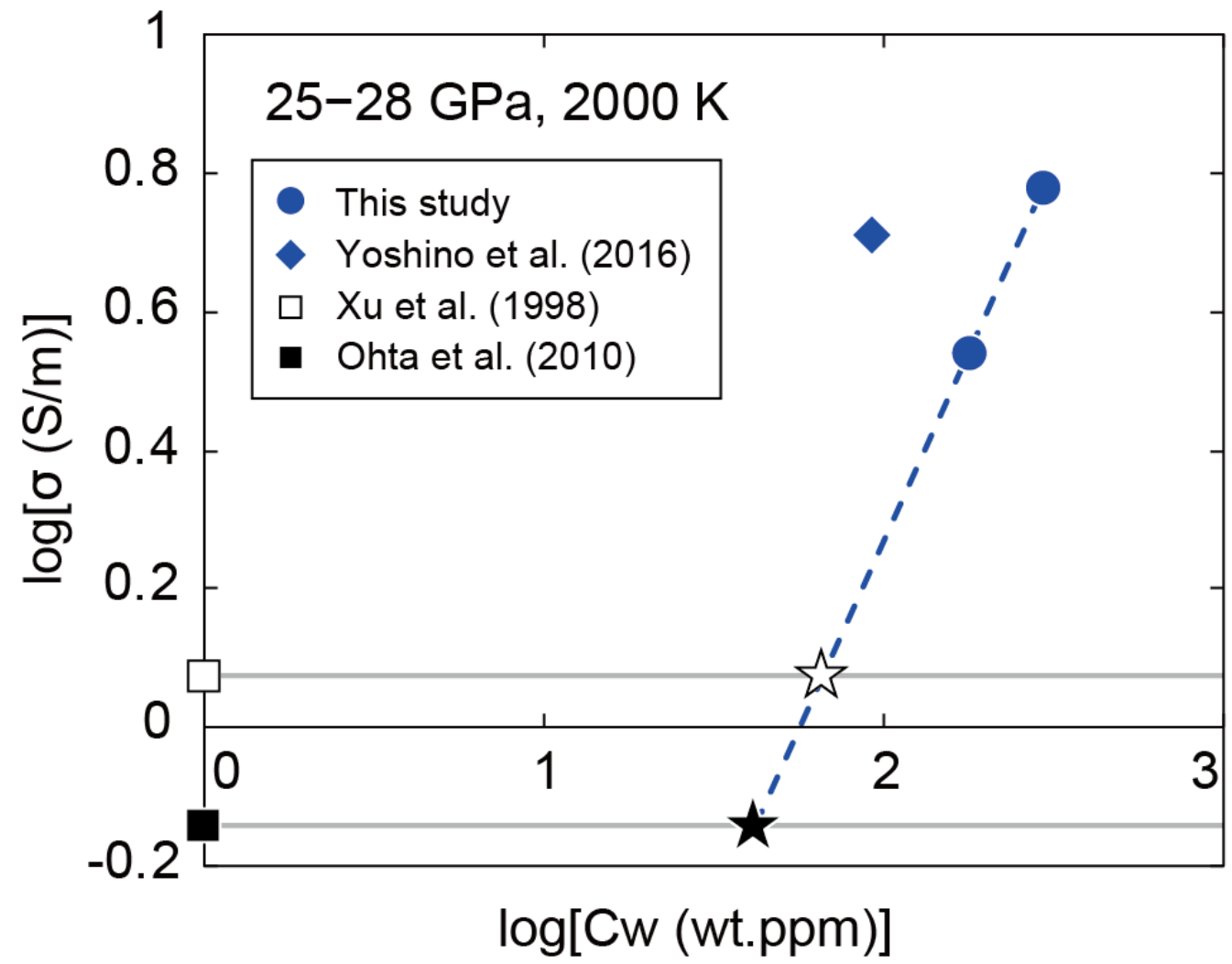

Supplementary Fig. 3 | Electrical conductivity of bridgmanite as a function of water content at 25−28 GPa, 2000 K. Blue circles show the electrical conductivity of bridgmanite containing 293 and 177 wt. ppm H_2O (this study), and the blue diamond shows 92 wt. ppm H_2O-bearing bridgmanite[13]. Black open and filled squares represent the electrical conductivity of dry (Fe,Al)-bearing bridgmanite[4] and dry pyrolite[2], respectively. Black open and filled stars indicate the estimated water contents at which proton conduction in hydrous bridgmanite equals the small-polaron conductivity of dry (Fe,Al)-bearing bridgmanite[4] and dry pyrolite[2], respectively, based on linear extrapolation of our data. The crossover occurs at 67 ± 18 wt. ppm H_2O when using dry (Fe,Al)-bearing bridgmanite[4] as the reference and at 40 ± 11 wt. ppm H_2O when using dry pyrolite[2] as the reference. These nominal crossover values suggest that bridgmanite with less than ~40–70 wt. ppm H_2O will predominantly exhibit small-polaron conduction rather than proton conduction. Therefore, this range represents an approximate lower limit of water content that can be resolved by comparing laboratory electrical conductivity measurements with geophysical observations.

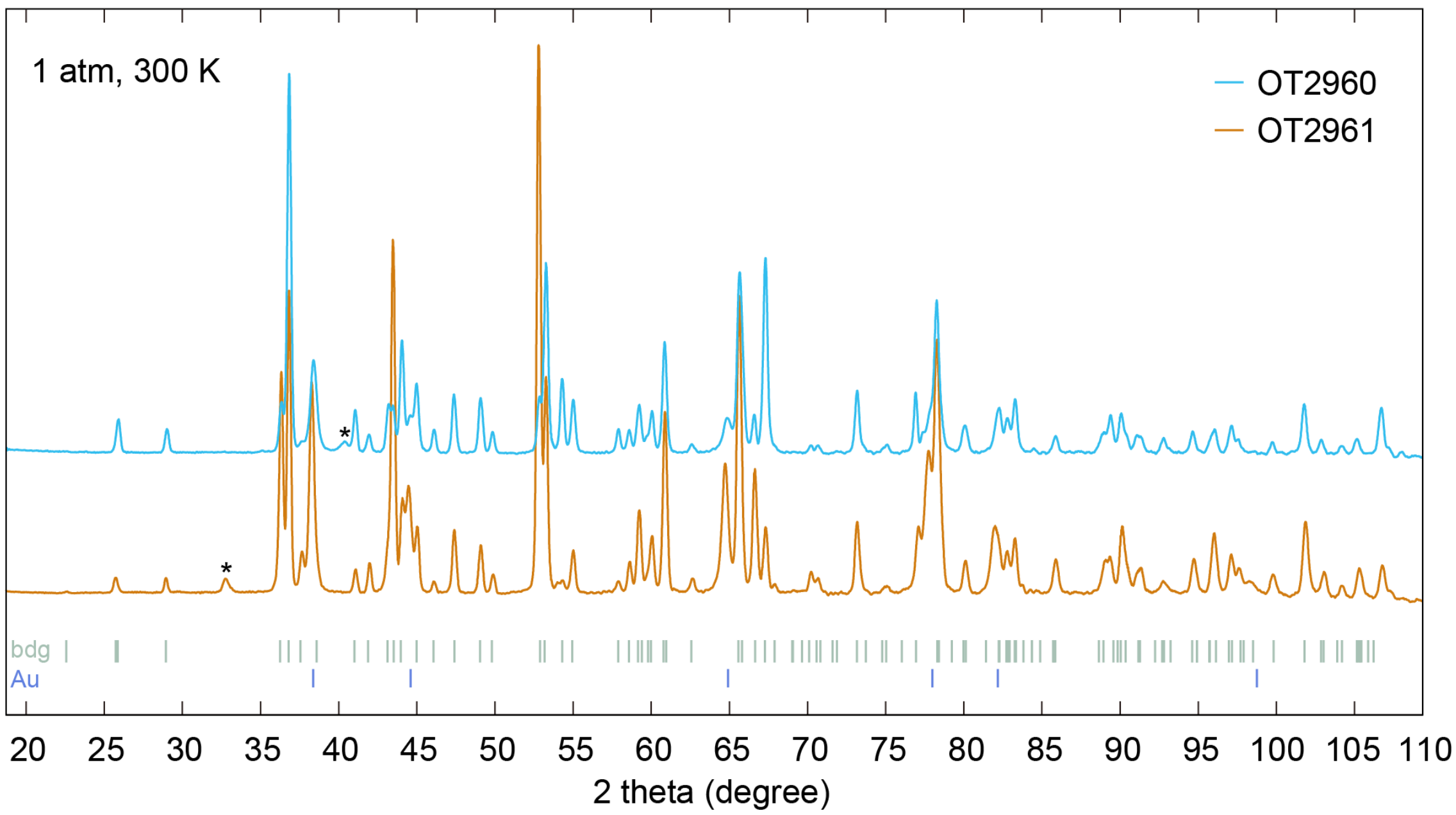

Supplementary Fig. 4 | XRD results of hydrous bridgmanite samples at ambient pressure. Light blue and brown patterns indicate the XRD results of samples synthesized in runs OT2960 and OT2961, respectively. The green and dark blue lines indicate the diffraction peak locations of bridgmanite and Au, respectively. All observed peaks, except for the unidentified peak marked with an asterisk (which does not match potential minor phases such as brucite or corundum), correspond to the diffraction patterns of bridgmanite and gold.

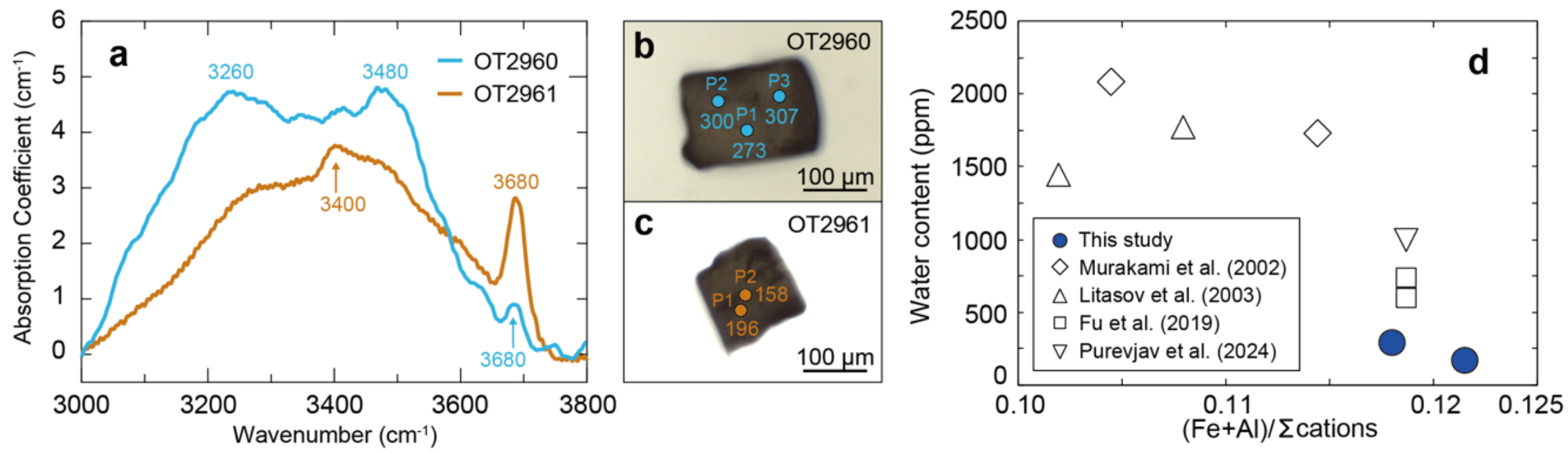

Supplementary Fig. 5 | FTIR results of the hydrous bridgmanite sample. (a) Unpolarized FTIR spectrum of the hydrous bridgmanite samples. Light blue and brown patterns indicate the FTIR results of samples synthesized in runs OT2960 and OT2961, respectively. FTIR peak at 3680 cm^{-1} is from a trace amount of brucite[14]. (**b, c),** microscopic images of hydrous bridgmanite samples synthesized in runs OT2960 and OT2961, respectively. Circles indicate positions where we conducted FTIR measurements. Numbers next to the circles indicate estimated water concentrations in wt. ppm H_2O. **(d)** Comparison of water content in bridgmanite with pyrolitic composition based on FTIR analysis as a function of (Fe+Al)/(Mg+Fe+Al+Si). Diamond, triangle, square, and reverse triangle symbols indicate the water solubility in (Fe,Al)-bearing bridgmanite with a pyrolitic composition, as reported by Murakami et al.[15], Litasov et al.[16], Fu et al.[17], and Purevjav et al.[18], respectively. Previous studies on H_2O solubility in (Fe,Al)-bearing bridgmanite with either higher[16] or lower[19] Fe,Al content than pyrolitic composition is below 50 ppm, which falls out of the range and is not plotted here.

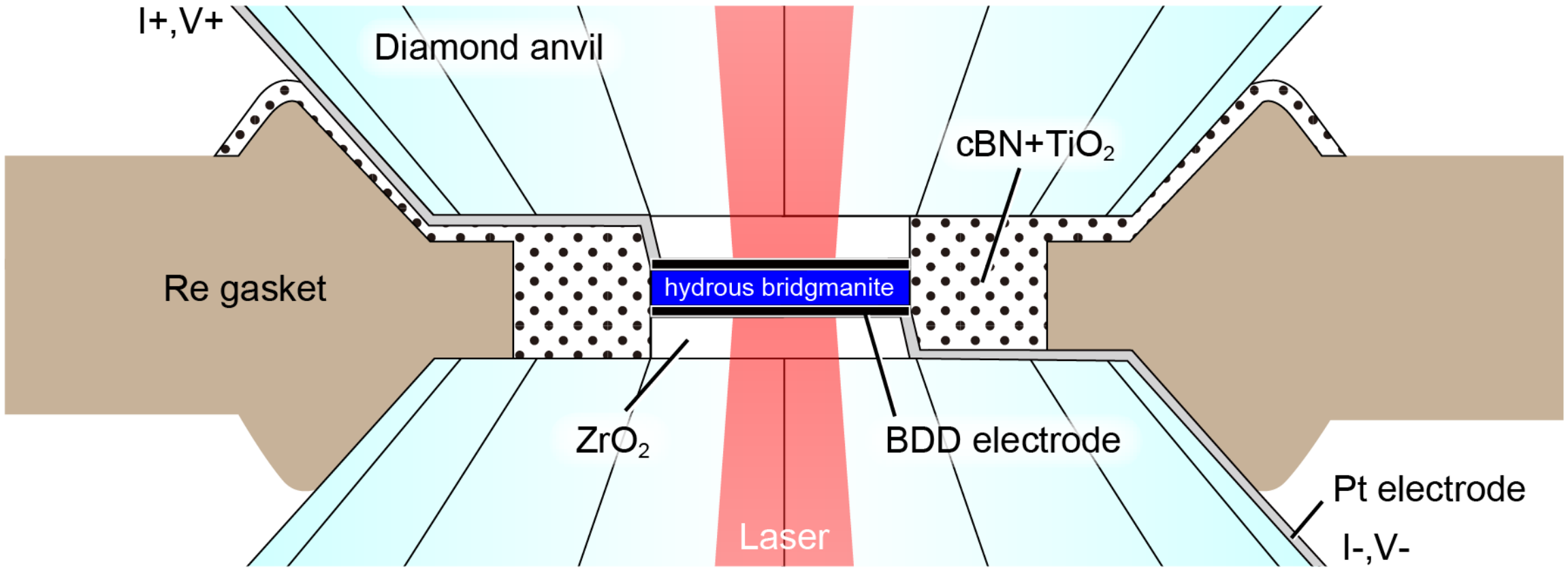

Supplementary Fig. 6 | Schematic illustration of the cell assembly for electrical conductivity measurements. Pt electrode was connected to a copper wire and a source meter (or a free response analyzer).

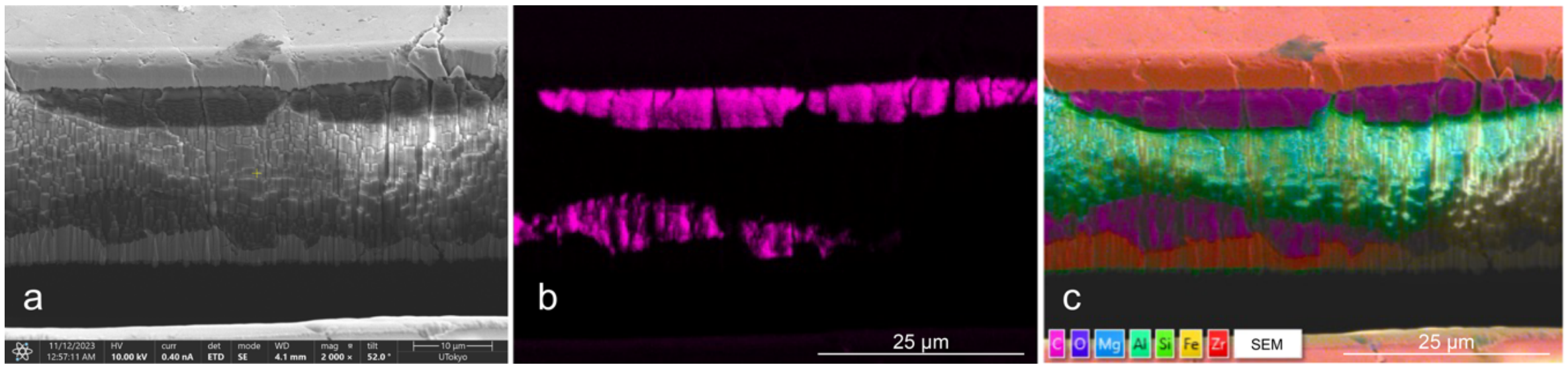

Supplementary Fig. 7 | Cross section of a sample recovered from run #4 for 177 wt. ppm H_2O sample. (a) SEM image, (b) EDS elemental mapping of carbon, and (c) EDS elemental mapping of C, O, Mg, Al, Si, Fe, and Zr. Note that figures were captured from an oblique direction of 52 degrees to the sample stage, and thus the apparent thickness shown here is different from the actual sample thickness given in Table 1.

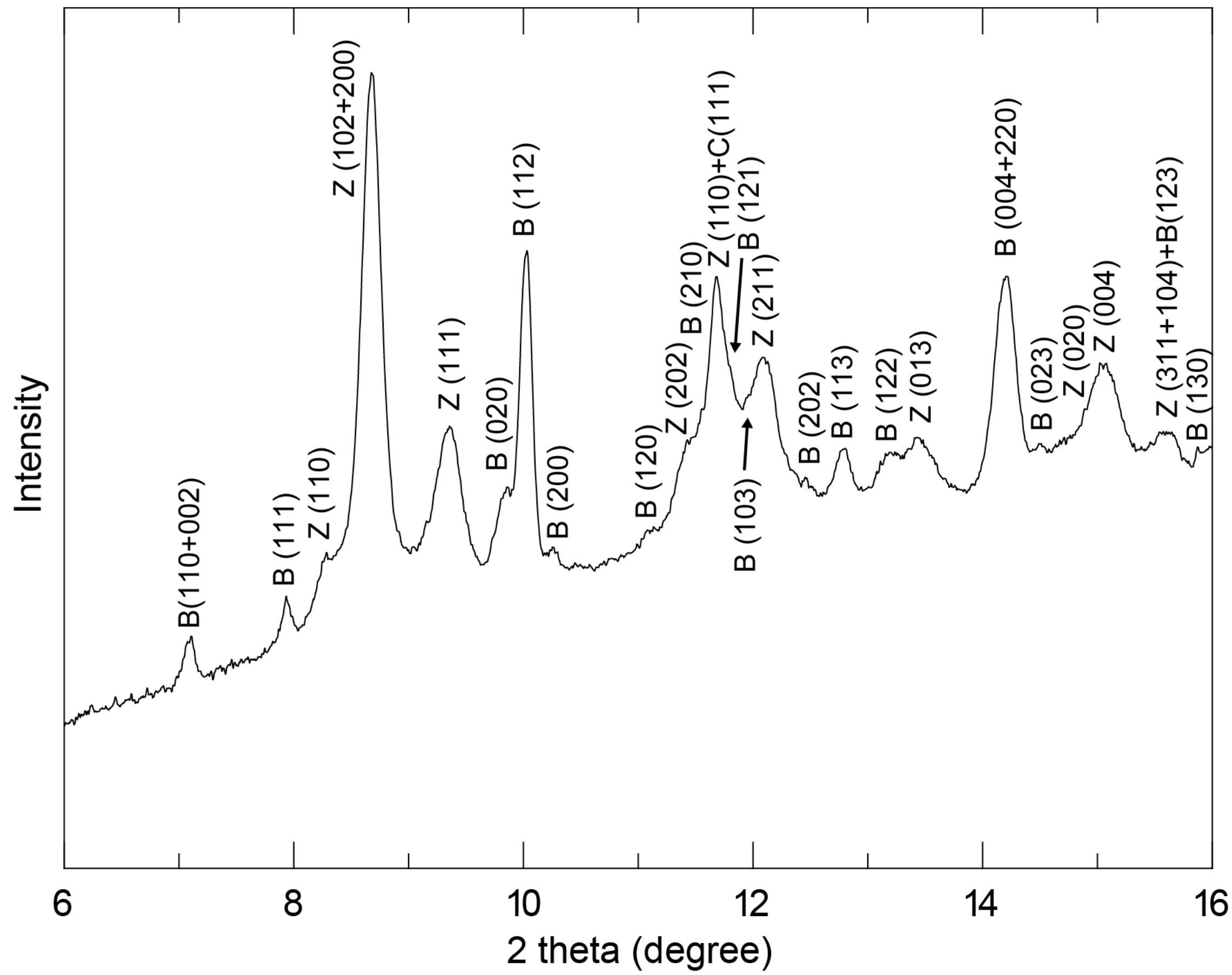

Supplementary Fig. 8 | X-ray diffraction patterns collected in run #1 for 177 wt. ppm H_2O-bearing bridgmanite sample after the heating experiment. B, Z, and C represent the diffraction peaks of bridgmanite, zirconia, and diamond, respectively. We did not observe any phases other than the bridgmanite sample, zirconia thermal insulator, and boron-doped diamond electrodes. No evidence of other hydrous phases, such as brucite or pure H_2O, was observed.

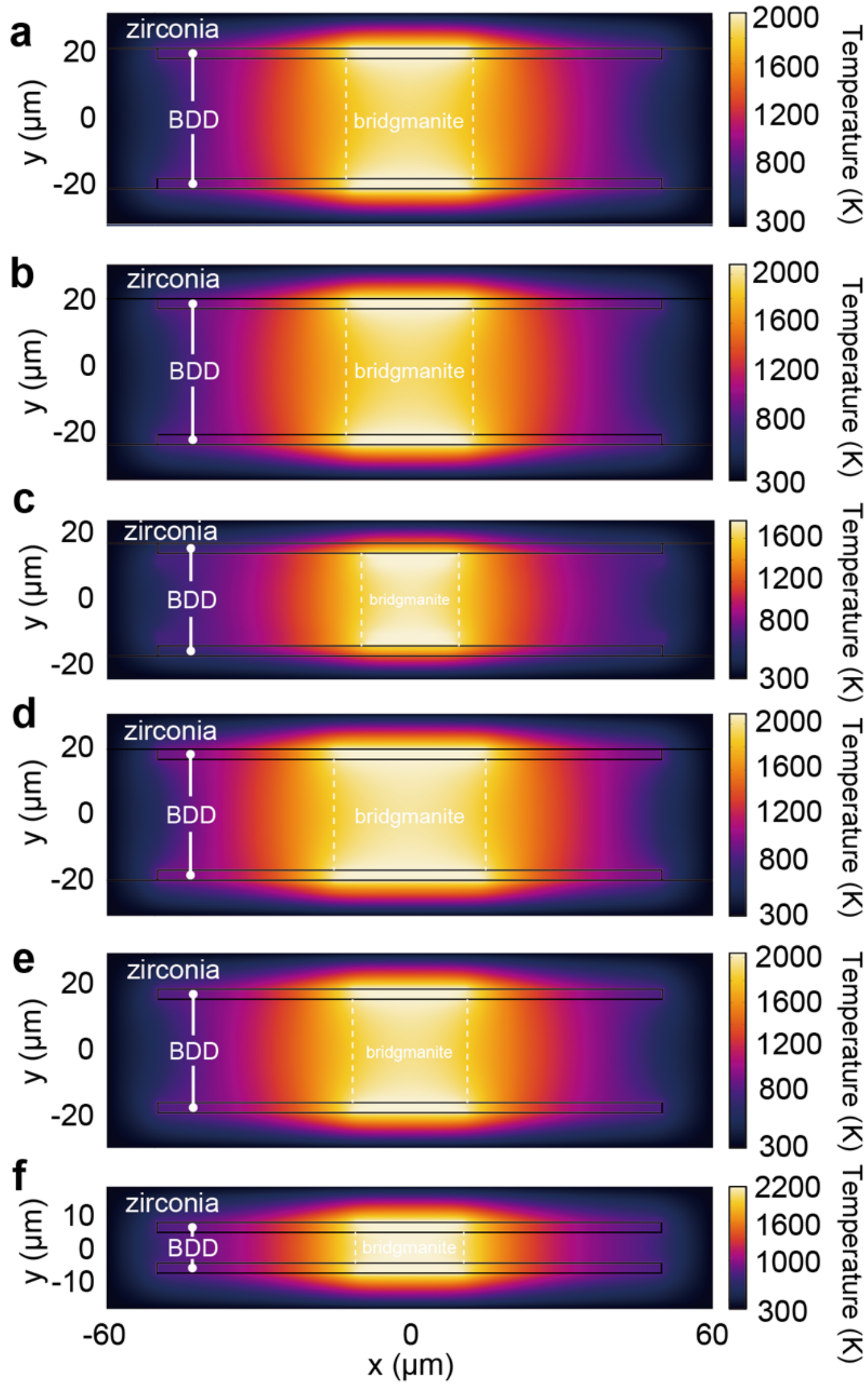

Supplementary Fig. 9 | Cross-sectional temperature distributions of hydrous bridgmanite simulated in 3D finite element method. (a−d) 177 wt. ppm H_2O sample temperature distributions in run #1 at 35 GPa heated up to 1890 K (a), run #2 at 38 GPa heated to 1960 K (b), run #3 at 37 GPa heated to 1530 K (c), and run #4 at 43 GPa heated to 2010 K (c), respectively. (**d, e)** 293 wt. ppm H_2O sample temperature distributions in run #1 at 35 GPa heated up to 1970 K (a) and run #2 at 47 GPa heated to 2210 K, respectively. A homogeneous circular area *S* with a diameter of the laser spot size was considered on both sample surfaces, and the applied heat was conducted to the sample and surrounding 10 μm-thick zirconia layers. The spatially averaged temperature within a cylindrical region, defined by circular top and bottom surfaces with area *S* and a height corresponding to the sample thickness, as indicated by white dashed lines, is considered as the experimental sample temperature in this study.

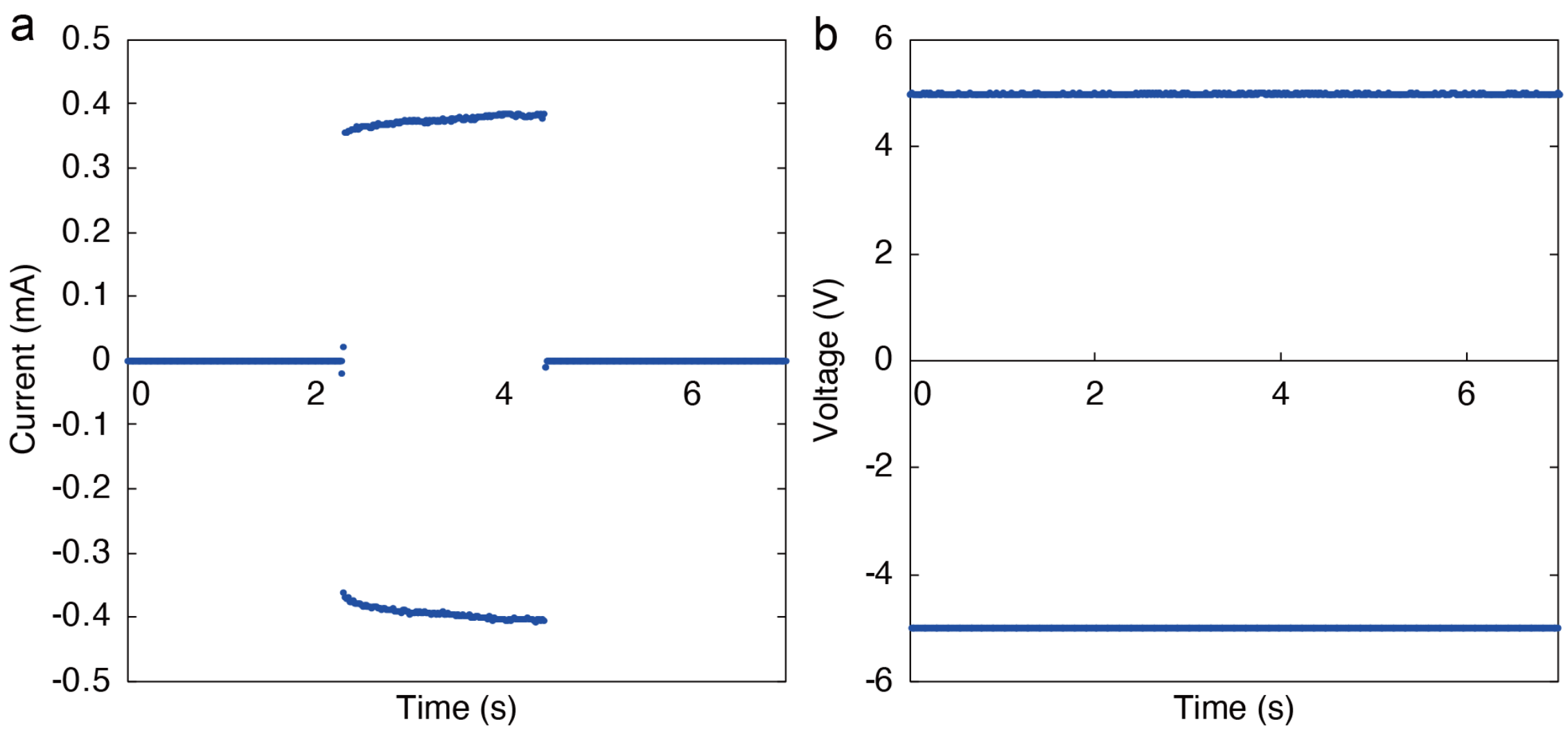

Supplementary Fig. 10 | Time evolution of (a) current and (b) voltage during heating to 2020 K at 47 GPa in run #2 for the 293 wt. ppm H_2O-bearing bridgmanite sample.

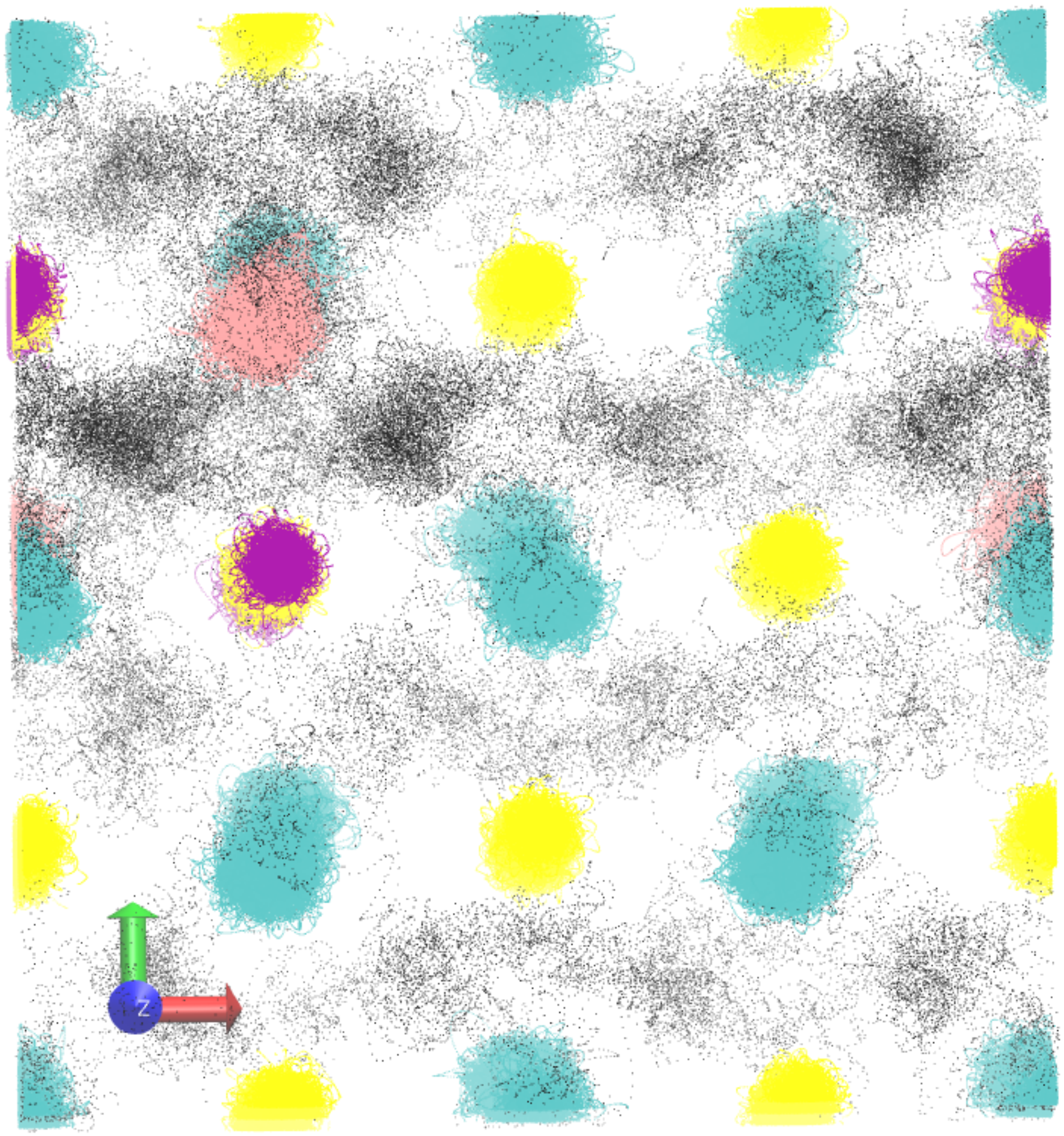

Supplementary Fig. 11 | Proton diffusion trajectory shown as black dashed lines from a 25 ps snapshot from an MD simulation for Fe,Al-bearing bridgmanite at 2500 K and 35 GPa using an orthorhombic 81-atom simulation with composition of $(Mg_{0.875}Fe^{2+}_{0.0625}Fe^{3+}_{0.0625})(Al_{0.125}Si_{0.875})O_3$. Yellow, purple, and pink colors represent Si, Al, and Fe, respectively. The oxygen atoms are omitted for clarity. The trajectories are drawn using VMD[20].

Table S1. Fitting results on σ of hydrous bridgmanite.

H_2O (ppm)	run#	P (GPa)	ΔH (eV)	$\log[\sigma_0$(S/m)]
177	1	35(4)	0.68(1)	2.06(4)
	2	37(4)	0.68(1)	1.99(3)
	3	37(4)	0.69(1)	2.08(9)
	4	44(4)	0.75(2)	2.07(5)
293	1	35(4)	0.68(2)	2.31(6)
	2	47(5)	0.47(1)	1.51(2)
			0.46(0)*	1.48(0)*

*: values when the room temperature datum was included in the fitting.

Table S2. Fitting results on diffusion coefficient of hydrous bridgmanite in this study.

H_2O (ppm)	run#	P (GPa)	ΔH (eV)	$\log[D_0(S/m)]$
177	1	35	0.82(2)	-4.96(5)
	2	37	0.82(1)	-5.04(3)
	3	37	0.79(2)	-5.02(6)
	4	44	0.89(1)	-4.94(4)
293	1	47	0.58(1)	-5.83(2)
	2		0.51(0)*	-6.00(2)*
2135**	computational	35	0.48(10)	-6.71(22)

*: values when the room temperature datum was included in the fitting.

**: H_2O content in *ab initio* calculation performed in this study.

Supplementary Discussion

The isotopic similarity between the bulk silicate Earth and enstatite chondrites suggests that a significant proportion of Earth's building blocks originated from enstatite chondrites[21–23]. Enstatite chondrites are traditionally considered anhydrous[24]. Consequently, Earth's water is generally attributed to the late-stage addition of a small fraction of hydrated materials, such as carbonaceous chondrite meteorites. To account for the observed excess of highly siderophile elements, including rare earth elements, it has been hypothesized that ~1−4% of primitive meteoritic material was delivered after core formation, a process known as the 'late-veneer' hypothesis[25–27]. Carbonaceous chondrites are considered the strongest candidates for this late-veneer material[26], which contains a high water content of ~10 wt.% H_2O[28]. Such water supplied at the Earth's surface may not have effectively hydrated the lower mantle. Although the present Earth exhibits whole-mantle convection[29] or a transitional stage from earlier double-layer convection[30,31], it is suggested that early Earth underwent double-layer convection due to higher internal temperatures and elevated Rayleigh number[32]. Such a configuration could have inhibited chemical exchange between the upper and lower mantle, potentially preventing water provided at the Earth's surface from hydrating the lower mantle. This scenario could account for the dry lower mantle bridgmanite observed in this study, when assuming that enstatite chondrites are anhydrous[24]. Nevertheless, recent studies have demonstrated that enstatite chondrites could contain moderate amounts of water, ranging ~0.1−1.0 wt.% H_2O, depending on their metamorphic grade[33]. In this case, bridgmanite crystallized from magma ocean would be expected to incorporate water up to its solubility limit.

As discussed in the main manuscript, hydrogen is highly siderophile at high *P*-*T* conditions[35–37], so a significant amount of hydrogen may have partitioned from bridgmanite to metallic iron in the lower mantle. In this scenario, iron reacts with H_2O to form wüstite and iron hydride (FeH_x) through the reaction: $3Fe + H_2O \rightarrow FeO + 2FeH$[36]. Assuming that lower mantle bridgmanite was initially water-saturated with the reported maximum water solubility of approximately 4000 ppm H_2O[38], and assuming iron consumes all the water in bridgmanite, iron in isolated pockets will form an iron hydride with 37.5 mol.% hydrogen

(equivalent to x=0.6). Moreover, the FeO produced in this reaction could oxidize the surrounding mantle[36], potentially contributing to the secular oxidation of the Earth's mantle[39]. Although FeH_x has a significantly lower melting temperature than pure iron[35,40], the melting temperature of $FeH_{x(0<x<1)}$[41] remains higher than the present-day lower mantle geotherm, indicating that FeH_x formed in the lower mantle would remain solid and would be trapped in the grain boundaries (Supplementary Fig. 12).

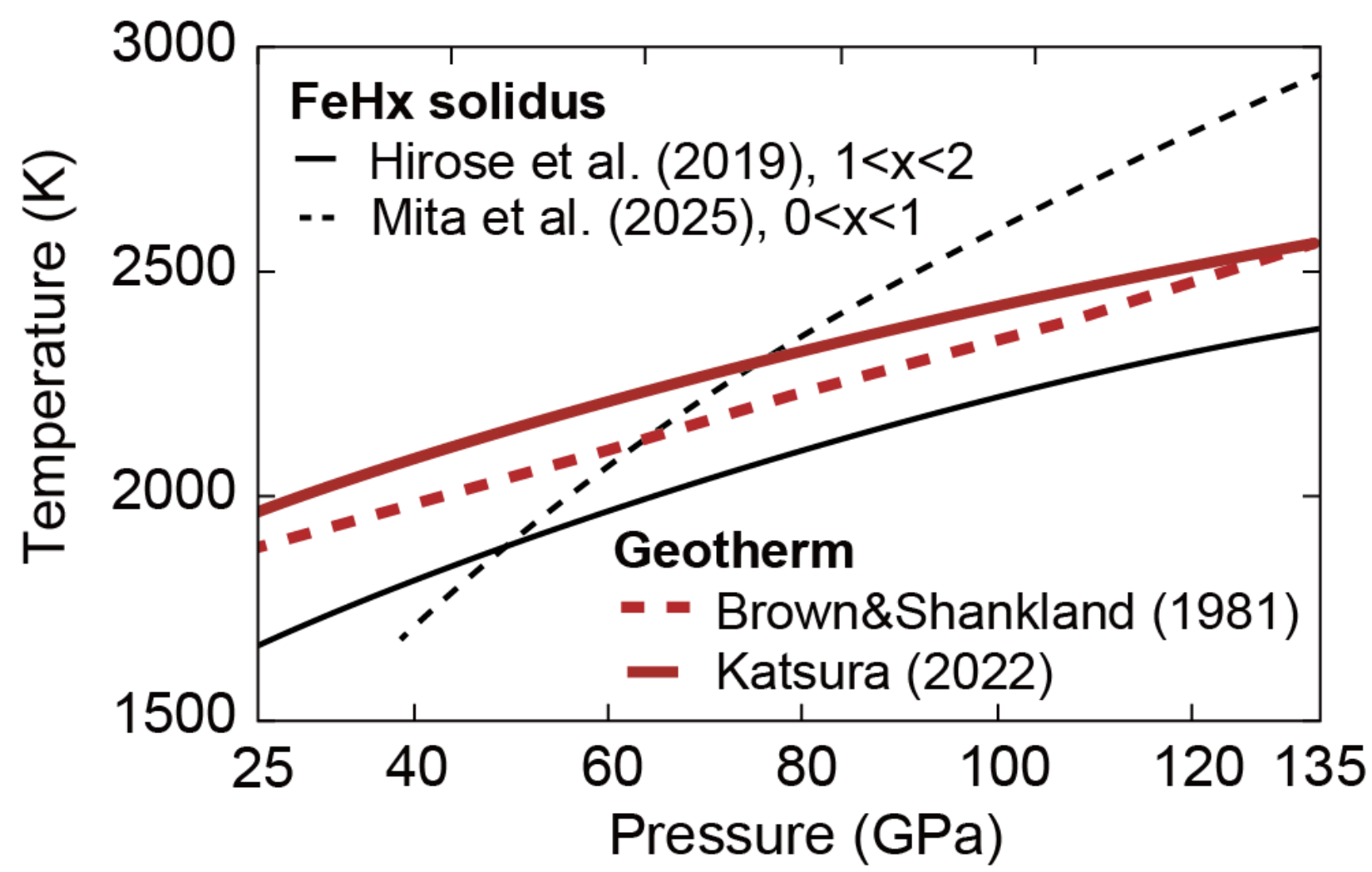

Supplementary Fig. 12 | Comparison between mantle geotherm and solidus of FeHx in the Earth's mantle. Solid and broken curves and indicate the solidus of FeHx with 1<x<2 (Ref[42]) and 0<x<1 (Ref[41]), respectively. Red solid and broken lines indicate mantle geotherm by Brown & Shankland[43] and Katsura[1].

However, since the early Earth was hotter than it is today, iron hydride formed under such conditions could have been molten, potentially percolating through the lower mantle and eventually reaching the core-mantle boundary, as supported by studies on wetting angles between molten iron and bridgmanite[44,45]. As a result, some metallic iron formed through disproportionation reactions in lower mantle bridgmanite may have migrated to the core, possibly transporting rare earth elements and other siderophile elements from the lower mantle. This process could have influenced the geochemical distribution of siderophile trace elements in the Earth's lower mantle[46,47].

The partition coefficient of H_2O between bridgmanite and metallic iron has yet to be experimentally constrained. This parameter is critical, as it determines the extent to which hydrogen can be incorporated into metallic iron, thereby influencing its melting behavior and the likelihood that it could have existed in molten form under the higher temperatures of the early Earth's lower mantle. Also, although the present study demonstrates that lower mantle bridgmanite is geophysically dry, the electrical conductivity of dry, pyrolitic bridgmanite has not yet been directly measured under lower mantle conditions using DAC techniques. Future investigations should aim to determine whether dry, pyrolitic bridgmanite alone can explain the observed depth-dependent conductivity structure of the lower mantle.

Supplementary References

1. Katsura, T. A Revised Adiabatic Temperature Profile for the Mantle. *JGR Solid Earth* **127**, e2021JB023562 (2022).
2. Ohta, K. *et al.* Electrical conductivities of pyrolitic mantle and MORB materials up to the lowermost mantle conditions. *Earth and Planetary Science Letters* **289**, 497–502 (2010).
3. Sinmyo, R., Pesce, G., Greenberg, E., McCammon, C. & Dubrovinsky, L. Lower mantle electrical conductivity based on measurements of Al, Fe-bearing perovskite under lower mantle conditions. *Earth and Planetary Science Letters* **393**, 165–172 (2014).
4. Xu, Y., McCammon, C. & Poe, B. T. The Effect of Alumina on the Electrical Conductivity of Silicate Perovskite. *Science* **282**, 922–924 (1998).
5. Trubowitz, C. *et al.* The Electrical Conductivity of the Lower Mantle From High-Pressure/High-Temperature Measurements of Pyrolite and Ten Years of Geomagnetic Observations. *JGR Solid Earth* **131**, e2025JB032837 (2026).
6. Olsen, N. Long-period (30 days-1 year) electromagnetic sounding and the electrical conductivity of the lower mantle beneath Europe. *Geophys. J. Int.* **138**, 179–187 (1999).
7. Kuvshinov, A. & Olsen, N. A global model of mantle conductivity derived from 5 years of CHAMP, Ørsted, and SAC-C magnetic data. *Geophys. Res. Lett.* **33**, L18301 (2006).
8. Velímský, J. Electrical conductivity in the lower mantle: Constraints from CHAMP satellite data by time-domain EM induction modelling. *Phys. Earth Planet. Inter.* **180**, 111–117 (2010).

9. Civet, F. & Tarits, P. Analysis of magnetic satellite data to infer the mantle electrical conductivity of telluric planets in the solar system. *Planet. Space Sci.* **84**, 102–111 (2013).

10. Püthe, C. & Kuvshinov, A. Determination of the 1-D distribution of electrical conductivity in Earth's mantle from Swarm satellite data. *Earth Planet Sp* **65**, 1233–1237 (2013).

11. Civet, F., Thébault, E., Verhoeven, O., Langlais, B. & Saturnino, D. Electrical conductivity of the Earth's mantle from the first Swarm magnetic field measurements. *Geophys. Res. Lett.* **42**, 3338–3346 (2015).

12. Püthe, C., Kuvshinov, A., Khan, A. & Olsen, N. A new model of Earth's radial conductivity structure derived from over 10 yr of satellite and observatory magnetic data. *Geophys. J. Int.* **203**, 1864–1872 (2015).

13. Yoshino, T., Kamada, S., Zhao, C., Ohtani, E. & Hirao, N. Electrical conductivity model of Al-bearing bridgmanite with implications for the electrical structure of the Earth's lower mantle. *Earth Planet. Sci. Lett.* **434**, 208–219 (2016).

14. Bolfan-Casanova, N., Keppler, H. & Rubie, D. C. Water partitioning between nominally anhydrous minerals in the MgO–SiO2–H2O system up to 24 GPa: implications for the distribution of water in the Earth's mantle. *Earth and Planetary Science Letters* **182**, 209–221 (2000).

15. Murakami, M., Hirose, K., Yurimoto, H., Nakashima, S. & Takafuji, N. Water in Earth's Lower Mantle. *Science* **295**, 1885–1887 (2002).

16. Litasov, K. *et al.* Water solubility in Mg-perovskites and water storage capacity in the lower mantle. *Earth Planet. Sci. Lett.* **211**, 189–203 (2003).

17. Fu, S. *et al.* Water Concentration in Single-Crystal (Al,Fe)-Bearing Bridgmanite Grown From the Hydrous Melt: Implications for Dehydration Melting at the Topmost Lower Mantle. *Geophys. Res. Lett.* **46**, 10346–10357 (2019).

18. Purevjav, N. *et al.* Hydrogen incorporation mechanism in the lower-mantle bridgmanite. *Am. Mineral.* **109**, 1036–1044 (2024).

19. Liu, Z. *et al.* Bridgmanite is nearly dry at the top of the lower mantle. *Earth Planet. Sci. Lett.* **570**, 117088 (2021).

20. Humphrey, W., Dalke, A. & Schulten, K. VMD: Visual molecular dynamics. *J. Mol. Graph.* **14**, 33–38 (1996).

21. Dauphas, N. The isotopic nature of the Earth's accreting material through time. *Nature* **541**, 521–524 (2017).

22. Javoy, M. *et al.* The chemical composition of the Earth: Enstatite chondrite models. *Earth Planet. Sci. Lett.* **293**, 259–268 (2010).

23. Warren, P. H. Stable-isotopic anomalies and the accretionary assemblage of the Earth and Mars: A subordinate role for carbonaceous chondrites. *Earth Planet. Sci. Lett.* **311**, 93–100 (2011).

24. Robert, F., Javoy, M., Halbout, J., Dimon, B. & Merlivat, L. Hydrogen isotope abundances in the solar system. Part II: Meteorites with terrestrial-like ratio. *Geoch. Cosm. Act.* **51**, 1807–1822 (1987).

25. Albarède, F. Volatile accretion history of the terrestrial planets and dynamic implications. *Nature* **461**, 1227–1233 (2009).

26. Fischer-Gödde, M. *et al.* Ruthenium isotope vestige of Earth's pre-late-veneer mantle preserved in Archaean rocks. *Nature* **579**, 240–244 (2020).

27. Wang, Z. & Becker, H. Ratios of S, Se and Te in the silicate Earth require a volatile-rich late veneer. *Nature* **499**, 328–331 (2013).

28. King, A. J., Solomon, J. R., Schofield, P. F. & Russell, S. S. Characterising the CI and CI-like carbonaceous chondrites using thermogravimetric analysis and infrared spectroscopy. *Earth Planet Sp* **67**, 198 (2015).

29. Fukao, Y. Seismic Tomogram of the Earth's Mantle: Geodynamic Implications. *Science* **258**, 625–630 (1992).

30. Christensen, U. R. & Yuen, D. A. The interaction of a subducting lithospheric slab with a chemical or phase boundary. *J. Geophys. Res.* **89**, 4389–4402 (1984).

31. Nakakuki, T., Sato, H. & Fujimoto, H. Interaction of the upwelling plume with the phase and chemical boundary at the 670 km discontinuity: Effects of temperature-dependent viscosity. *Earth Planet. Sci. Lett.* **121**, 369–384 (1994).

32. Ida, S. & Maruyama, S. Drastic change of mantle convection from two layered to whole mantle convection at 1.9 Ga. (1992).

33. Piani, L. *et al.* Earth's water may have been inherited from material similar to enstatite chondrite meteorites. *Science* **369**, 1110–1113 (2020).

34. Frost, D. J. *et al.* Experimental evidence for the existence of iron-rich metal in the Earth's lower mantle. *Nature* **428**, 409–412 (2004).

35. Hirose, K. *et al.* Hydrogen Limits Carbon in Liquid Iron. *Geophysical Research Letters* **46**, 5190–5197 (2019).

36. Okuchi, T. Hydrogen partitioning into molten iron at high pressure: Implications for earth's core. *Science* **278**, 1781–1784 (1997).

37. Tagawa, S. *et al.* Experimental evidence for hydrogen incorporation into Earth's core. *Nat. Commun.* **12**, 2588 (2021).

38. Murakami, M., Hirose, K., Yurimoto, H., Nakashima, S. & Takafuji, N. Water in Earth ' s Lower Mantle. **295**, 1885–1888 (2002).

39. Nicklas, R. W. *et al.* Secular mantle oxidation across the Archean-Proterozoic boundary: Evidence from V partitioning in komatiites and picrites. *Geochimica et Cosmochimica Acta* **250**, 49–75 (2019).

40. Sakamaki, K. *et al.* Melting phase relation of FeH up to 20 GPa: Implication for the temperature of the Earth's core. *Physics of the Earth and Planetary Interiors* **174**, 192–201 (2009).

41. Mita, S., Tagawa, S., Hirose, K. & Ikuta, N. Fe-FeH Eutectic Melting Curve and the Estimates of Earth's Core Temperature and Composition. *JGR Solid Earth* **130**, e2024JB029283 (2025).

42. Hirose, K. *et al.* Hydrogen Limits Carbon in Liquid Iron. *Geophys. Res. Lett.* **46**, 5190–5197 (2019).

43. Brown, J. M. & Shankland, T. J. Thermodynamic parameters in the Earth as determined from seismic profiles. *Geophys J Int* **66**, 579–596 (1981).

44. Shi, C. Y. *et al.* Formation of an interconnected network of iron melt at Earth's lower mantle conditions. *Nature Geoscience* **6**, 971–975 (2013).

45. Shannon, M. C. & Agee, C. B. Percolation of core melts at lower mantle conditions. *Science* **280**, 1059–1061 (1998).

46. O'Neill, H. S. C. The origin of the moon and the early history of the earth-A chemical model. Part 1: The moon. *Geochimica et Cosmochimica Acta* **55**, 1135–1157 (1991).

47. Kimura, K., Lewis, R. S. & Anders, E. Distribution of gold and rhenium between nickel-iron and silicate melts: implications for the abundance of siderophile elements on the Earth and Moon. *Geochimica et Cosmochimica Acta* **38**, 683–701 (1974).
