# Supplementary material for: Geophysical evidence of a nearly dry bridgmanite in the Earth’s lower mantle
Source: Nat Commun. 2026 Aug 12;17:9684. doi: 10.1038/s41467-026-76621-1 (PMC13558727; doi:10.1038/s41467-026-76621-1)
Supplement: Supplementary file 2 [file 41467_2026_76621_MOESM2_ESM.pdf]

nature portfolio

Peer Review File

Geophysical evidence of a nearly dry bridgmanite in the Earth's lower mantle

Corresponding Author: Dr Yoshiyuki Okuda

This file contains all reviewer reports in order by version, followed by all author rebuttals in order by version.

Version 0:

Reviewer comments:

Reviewer #1

(Remarks to the Author)
Okuda et al. measured the resistance of bridgmanite containing 177 and 293 wt. ppm H_2O and found that water significantly reduces the resistance of bridgmanite. In other words, structural water increases the electrical conductivity (EC) of bridgmanite. By comparing their results with the observed conductivity of the lower mantle, the authors conclude that bridgmanite in the mid-lower mantle contains less than 40 wt. ppm H_2O.

I find this study interesting and potentially important for understanding the deep Earth. However, I have several concerns about the experimental methodology.If the authors can address the following points convincingly, I would recommend publication.

1. The authors measured the resistance of the sample at high temperature, rather than the impedance. The resistance measurement usually cannot estimate the contact resistance and polarization on the sample. Therefore, researchers prefer the impedance measurement to determine EC. How do the authors validate that their resistance measurements represent the intrinsic sample conductivity? Although they cite their methodological paper (Ref. 42), that study also employed impedance spectroscopy rather than direct resistance measurements.

2. The authors measured impedance at room temperature, and the EC derived from these data agrees with that extrapolated from the high-temperature resistance data. This agreement indeed supports the reliability of their approach. However, could the authors explain why impedance measurements were not performed at high temperature? Demonstrating consistency between impedance and resistance measurements under identical high-temperature conditions would provide stronger validation.

3. In Supplementary Figure 9, the current increases with time. Could the authors clarify whether this behavior results from experimental setup issues or from the sample not yet reaching thermal equilibrium?

4. Yoshino et al. (2016) used impedance spectroscopy and reported higher EC values than those obtained in the present study. Although their experiments were conducted at lower pressures, the pressure effect alone seems insufficient to explain the discrepancy (Supplementary Figure 2). It would strengthen the paper if the authors could discuss possible causes for this difference.

5. Judging from Supplementary Figure 5, the current path appears to be Pt electrode → Re gasket → BDD electrode → Pt electrode. Did the authors accidently omit the insulation on the left side of the setup?

6. In Supplementary Figure 6, the lower BDD electrode appears damaged. Does this affect the measurements? In addition, the sample thickness varies considerably. The authors' previous study (Ref. 42) achieved much better geometric control. Is this difference related to the mechanical properties difference between BDD and Pt? If I understand correctly, BDD is harder than Pt. Therefore, it should be easier to keep better geometry. I recognize that such experiments are technically demanding; if this represents the best achievable setup at the current stage, this is understandable.

7. It would be straightforward for comparison if authors can include their own EC measurement of dry bridgmanite from their previous study.

Minor Comments:

L126. 'high-temperature EC data extrapolated to room temperature' maybe easier for reader to change to 'room temperature EC data extrapolated from high-temperature data'.
L152. Why exponent of Cw is 1/3? Maybe add references?
L157. 'Previous studies'. Add references.
L213. '1-2 orders of magnitude'. Add references.

Reviewer #2

(Remarks to the Author)
I appreciate the work presented in this study and recognize the technical challenges involved in developing such an experimental protocol. However, there are several critical components missing from the manuscript that need to be addressed before it can be considered further.
The clearly defined tails observed in the impedance spectra indicate the formation of a secondary phase (s) during the course of the experiments. It is important to note that impedance spectra are highly sensitive to chemical and physical changes occurring within the sample; therefore, even minute deviations in spectral shape should not be left unaddressed. Furthermore, the evolution of the spectra with increasing temperature provides crucial information about the processes taking place in the sample, e.g. dehydration, melting, diffusion, reactions etc...
The clear deviation from ideal semicircular behavior suggests the formation of additional phase(s), possibly related to fluid or melt generation, or to chemical segregation. The authors should discuss this point in greater depth, particularly in relation to the temperature evolution of the impedance spectra. In addition, the frequency range should be extended to lower frequencies (0.1 or 0.001 Hz) to better capture and interpret the processes occurring in this regime. Such misinterpretations might otherwise lead to unrealistic conductivity estimates and compromise the quality of the discussion.
Moreover, the axial direction may not be the most appropriate orientation to capture chemical segregation that can occur in DAC experiments involving laser heating. The chemical analysis of the experimental run products should also be performed on a plane perpendicular to the axial direction to assess potential effects of temperature gradients.
Finally, the distance between the two electrodes appears to vary significantly. The authors should clarify how such deformations were accounted for during data analysis and conductivity calculations.
While the theoretical predictions provide a valuable and interesting dimension to the discussion, it is important that they do not substitute for a clear and detailed description of the data acquisition process and experimental protocols, as these aspects are essential to ensure the accuracy and reliability of the results.

Reviewer #3

(Remarks to the Author)
The authors measure the electrical conductivity (EC) of hydrous bridgmanite and use these results to infer water content in the lower mantle. This is an important topic, the high P–T EC measurements appear robust, and the manuscript is generally well written. But I have concerns on starting sample, water concentration, and how the inclusion affects EC results.
First, a few previous relevant studies need to be included in the paper:
Amulele et al. 2021, JGR Solid Earth, https://doi.org/10.1029/2021JB022222
Zhang et al. 2025, JGR Solid Earth, https://doi.org/10.1029/2024JB030403
Lu et al. 2025, Science, DOI: 10.1126/science.adx5883

The starting bridgmanite sample shows clear contamination from brucite inclusions (Supplementary Fig. 4; Lines 409-411). The authors report a lower water content compared to Ref. 33, but it is unclear whether this discrepancy reflects intrinsic solubility differences or results from brucite inclusions or precipitates formed during quenching, as discussed in Refs. 33, 38, and Amulele et al. (2021). If such inclusions formed during quenching, then in experiments heated to high P-T conditions, water potentially released from brucite would further hydrate bridgmanite. Moreover, recent results by Lu et al. (2025) show that water solubility in bridgmanite increases with temperature, suggesting that progressive hydration during heating is likely. Under these conditions, a sharp jump in conductivity, as proposed in Lines 420-422, may not occur.

The authors state that inclusions "do not affect EC as long as they are not interconnected" (Lines 417-418). However, the inclusions appear to be in direct contact with the bridgmanite matrix. Given that brucite has a lower electrical conductivity than bridgmanite (Line 419), such inclusions could bias the measurements toward lower EC values.

The authors use Paterson's calibration (Equation 6) to estimate water content. However, this calibration is known to be inappropriate for many silicate minerals, including bridgmanite, as discussed in Zhang et al. (2025). Application of this calibration may therefore introduce systematic errors in the estimated water concentrations, which directly affects the interpretation of the water effects on EC.

Please add error bars in Figs. 2-4. Also, what is the error bar for the threshold of 40 ppm H_2O (line 38 and elsewhere)?

Version 1:

Reviewer comments:

Reviewer #1

(Remarks to the Author)
The authors conducted the additional impedeance measurement at high temperature and the results are consisent with the resistance measurement.
In additon, the authors have added a run with Ir electrode to prove the effect of potential breakage of BDD electrode on the results are small.
Therefore, I have no further comments on the manuscript.

Reviewer #2

(Remarks to the Author)

Reviewer #4

(Remarks to the Author)
This manuscript presents an important new conductivity dataset for hydrous Fe-Al-bearing bridgmanite at lower-mantle conditions. The experimental work is valuable, the conductivity measurements appear technically sound, and the manuscript is generally well written. Comparing with geophysical conductivity observations has the potential to provide important constraints on water storage in the Earth's lower mantle. Overall, the authors have addressed the previous reviewers' comments well and the manuscript is now in much better shape. However, I still have concerns regarding the inferred <40 ± 11 ppm H2O constraint (Supplementary Fig. 3):

This estimate appears to be based on extrapolation from only two hydrous compositions (177 and 293 ppm H2O), which limits the ability to evaluate the conductivity–water relationship outside the measured range. More importantly, the manuscript itself suggests that conductivity may depend not only on water content but also on Fe-Al composition, as discussed in the comparison with Yoshino et al. If conductivity is sensitive to both water content and composition, it is unclear whether a conductivity–water relationship derived from a single bridgmanite composition can be uniquely applied to infer lower-mantle water contents.

In addition, the manuscript states that below ~40 ppm H2O, small-polaron conductivity exceeds proton conductivity. However, the ~40 ppm threshold appears to be defined by the intersection of an extrapolated hydrous bridgmanite conductivity trend and the conductivity of pyrolite reported by Ohta et al. Because pyrolite conductivity represents a bulk multiphase assemblage rather than dry bridgmanite itself, it is unclear whether this intersection uniquely defines the transition from proton-dominated to small-polaron-dominated conduction in bridgmanite or can be directly translated into a water-content threshold for lower-mantle bridgmanite.

Taken together, it is unclear how robustly a quantitative constraint of <40 ± 11 ppm H2O can be inferred from the available data. At a minimum, the assumptions underlying this estimate should be discussed more explicitly.

Version 2:

Reviewer comments:

Reviewer #4

(Remarks to the Author)
The authors have clarified the assumptions and limitations underlying the inferred lower-mantle water-content constraint and revised the discussion to better reflect the associated uncertainties. The interpretation of the quantitative estimate is now more appropriately presented.

I have no further comments and recommend the manuscript for publication.

Open Access This Peer Review File is licensed under a Creative Commons Attribution 4.0 International License, which permits use, sharing, adaptation, distribution and reproduction in any medium or format, as long as you give appropriate credit to the original author(s) and the source, provide a link to the Creative Commons license, and indicate if changes were made.
In cases where reviewers are anonymous, credit should be given to 'Anonymous Referee' and the source.
The images or other third party material in this Peer Review File are included in the article's Creative Commons license, unless indicated otherwise in a credit line to the material. If material is not included in the article's Creative Commons license and your intended use is not permitted by statutory regulation or exceeds the permitted use, you will need to obtain permission directly from the copyright holder.
To view a copy of this license, visit https://creativecommons.org/licenses/by/4.0/

Response to the comments by Reviewer #1 (reviewer comments in bold and our response in blue):

Okuda et al. measured the resistance of bridgmanite containing 177 and 293 wt. ppm H_2O and found that water significantly reduces the resistance of bridgmanite. In other words, structural water increases the electrical conductivity (EC) of bridgmanite. By comparing their results with the observed conductivity of the lower mantle, the authors conclude that bridgmanite in the mid-lower mantle contains less than 40 wt. ppm H_2O. I find this study interesting and potentially important for understanding the deep Earth. However, I have several concerns about the experimental methodology. If the authors can address the following points convincingly, I would recommend publication.

We thank the reviewer for their careful evaluation of our manuscript on static high-pressure and high-temperature measurements of the electrical conductivity of water using laser-heated diamond anvil cells. We appreciate the reviewer's positive assessment of the study's significance. We consider that the experimental approach, metrology, and data presentation are appropriate for the conditions investigated. Below, we address the specific concerns in detail and provide additional clarification of our methodology and data analysis.

1. The authors measured the resistance of the sample at high temperature, rather than the impedance. The resistance measurement usually cannot estimate the contact resistance and polarization on the sample. Therefore, researchers prefer the impedance measurement to determine EC. How do the authors validate that their resistance measurements represent the intrinsic sample conductivity? Although they cite their methodological paper (Ref. 42), that study also employed impedance spectroscopy rather than direct resistance measurements.

2. The authors measured impedance at room temperature, and the EC derived from these data agrees with that extrapolated from the high-temperature resistance data. This agreement indeed supports the reliability of their approach. However, could the authors explain why impedance measurements were not performed at high temperature? Demonstrating consistency between impedance and resistance measurements under identical high-temperature conditions would provide stronger validation.

We agree that it is important to perform impedance measurement to validate that our direct-resistance measurements represent the intrinsic sample conductivity. Based on the

reviewer's comments, we performed additional impedance measurements on the hydrous bridgmanite sample at 37–38 GPa and up to 1640 K of electrode surface temperature. We did not increase to higher temperatures since impedance measurements require much longer acquisition time that increases the risk of potential carbon contamination from diamond anvil and water loss from the sample to the environment. The spectra showed a semi-circle, together with a low-frequency straight-line feature. The semicircle reflects the intrinsic dielectric relaxation of the sample, with its diameter representing the bulk resistance, whereas the low-frequency linear component arises from electrode polarization associated with a double-layer formation at the blocking electrodes. Such a double-layer is commonly observed in impedance measurements of ionic conductors under blocking-electrode conditions, as discussed in the original and revised manuscript. We note that a diffusion-controlled (Warburg-type) response can also produce a linear feature in a Nyquist plot; however, this typically appears with an approximately 45° slope (corresponding to a constant-phase element with $\alpha \approx 0.5$). In contrast, the non-45° straight-line feature observed in our data is characteristic of electrode polarization rather than diffusion-limited transport. We further consulted with an electrochemistry expert (Prof. Rafał Jurczakowski, Warsaw University), who confirmed that the observed low-frequency response is most consistently explained by a double-layer formation associated with proton migration at the electrode–sample interface. This interpretation is further supported by the absence of additional semicircular features that would be expected from grain boundary contributions, as well as the lack of a low-frequency "hook" indicative of sample dehydration or fluid migration. Together, these observations indicate that the measured impedance response reflects the intrinsic bulk conductivity of the sample, while the low-frequency component arises from interfacial polarization effects.

Impedance measurements were performed using a HIOKI 3532-80, which operates in an LCR bridge configuration. Because impedance spectra require frequency scanning, data acquisition is relatively time-intensive; for example, at 1 Hz, a single data point requires approximately 1 s, and collecting a full spectrum typically requires heating durations exceeding 30 s (Okuda et al., 2022 RSI). In the initial experiments, high-temperature impedance measurements were not prioritized in order to limit heating duration and thereby reduce the potential for sample dehydration or chemical alteration. Following the reviewer's suggestion, we have now conducted additional experiments incorporating high *P*–*T* impedance measurements, and these new data have been included in the revised manuscript.

3. In Supplementary Figure 9, the current increases with time. Could the authors clarify whether this behavior results from experimental setup issues or from the sample not yet reaching thermal equilibrium?

We thank the reviewer for carefully examining our data. The observed increase in current with time is attributed to the approach to thermal equilibrium. In this dataset, thermal equilibrium is reached within approximately 1 s. Any temporal variation in resistance is accounted for in the reported uncertainty, as it was estimated from the standard deviation of the measured resistance values (lines 485–486 in the revised manuscript).

4. Yoshino et al. (2016) used impedance spectroscopy and reported higher EC values than those obtained in the present study. Although their experiments were conducted at lower pressures, the pressure effect alone seems insufficient to explain the discrepancy (Supplementary Figure 2). It would strengthen the paper if the authors could discuss possible causes for this difference.

The bridgmanite sample with 92 wt. ppm H_2O used in Yoshino et al. (2016) has a composition of ~9 mol% Fe and ~21 mol% Al, which differs from our samples containing ~11–12 mol% Fe and ~12–13 mol% Al.

Our calculations indicate that, relative to Al-poor compositions (e.g., ~6 mol% Al), bridgmanite containing combined Fe–Al contents of ~12.5 mol% exhibits proton diffusivity lower by ~0.1–0.2 log units. Although the individual effects of Fe and Al on proton transport remain difficult to fully disentangle, these results suggest that compositional differences, in addition to pressure, likely contribute to the discrepancy between the two studies.

In response to the reviewer's comment, we have expanded the discussion in the revised manuscript to address these compositional effects on page 8, lines 183–190 as follows.

"Yoshino et al.[20] reported that small polaron conduction was the most likely conduction mechanism, although they acknowledged that proton conduction could not be ruled out. The high EC observed in their study may partly reflect the negative pressure dependence of proton conductivity in hydrous bridgmanite, indicating that enhanced proton conduction could have contributed to the elevated EC in their measurement. In addition, their sample composition (~9 mol% Fe and ~21 mol% Al) differs from that of our samples (~11–12 mol% Fe and ~12–13 mol% Al), which may also have contributed to the discrepancy between the two studies."

5. Judging from Supplementary Figure 5, the current path appears to be Pt electrode → Re gasket → BDD electrode → Pt electrode. Did the authors accidently omit the insulation on the left side of the setup?

Thank you for pointing this out. We agree that the original figure contained an error in the depiction of the insulation on the left side of the setup. This has now been corrected in the revised figure (Supplementary Figure 6 in the revised manuscript), as shown below.

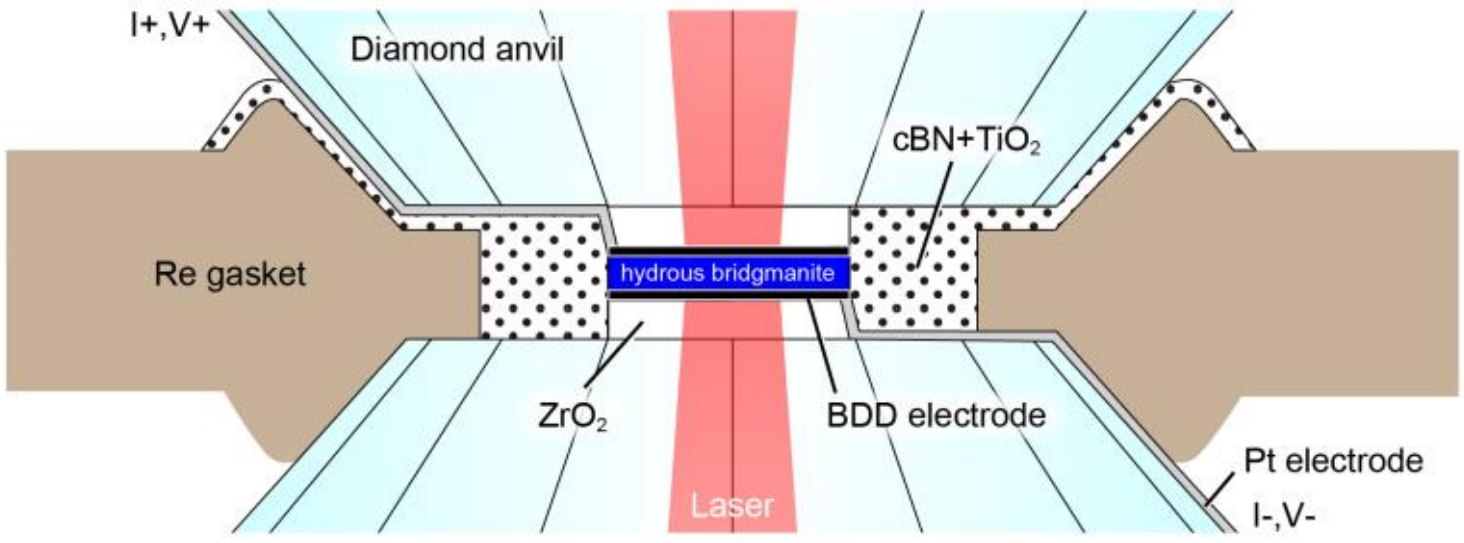

(Revised Supplementary Figure 5; included as Supplementary Figure 6 in the revised manuscript)

6. In Supplementary Figure 6, the lower BDD electrode appears damaged. Does this affect the measurements? In addition, the sample thickness varies considerably. The authors' previous study (Ref. 42) achieved much better geometric control. Is this difference related to the mechanical properties difference between BDD and Pt? If I understand correctly, BDD is harder than Pt. Therefore, it should be easier to keep better geometry. I recognize that such experiments are technically demanding; if this represents the best achievable setup at the current stage, this is understandable.

We thank the reviewer for this important observation. The apparent damage to the BDD electrode most likely occurred during sample recovery. Under high pressure, even at a few GPa, the sample region appears opaque because it is confined between BDD layers; however, upon decompression to ambient conditions, partial transmission of light is consistently observed. This suggests that the sample assembly expands during decompression, whereas the brittle BDD does not accommodate this expansion, leading to mechanical damage. However, we also considered the possibility that damage to the BDD electrode could influence the measurements, for example through the formation of microcracks at high *P*–*T* conditions. To evaluate this, we performed additional high *P*–*T* impedance measurements (run #3 for the 177 wt.% H_2O-bearing bridgmanite sample) using an iridium electrode, which is less brittle than BDD and exhibits improved structural integrity upon recovery. The SEM image below shows the cross-section of the recovered sample with iridium electrodes, revealing a smooth and flat electrode after recovery. The conductivity results obtained using the iridium electrode are consistent with

those obtained using BDD, indicating that damage observed in BDD does not affect the conductivity measurements.

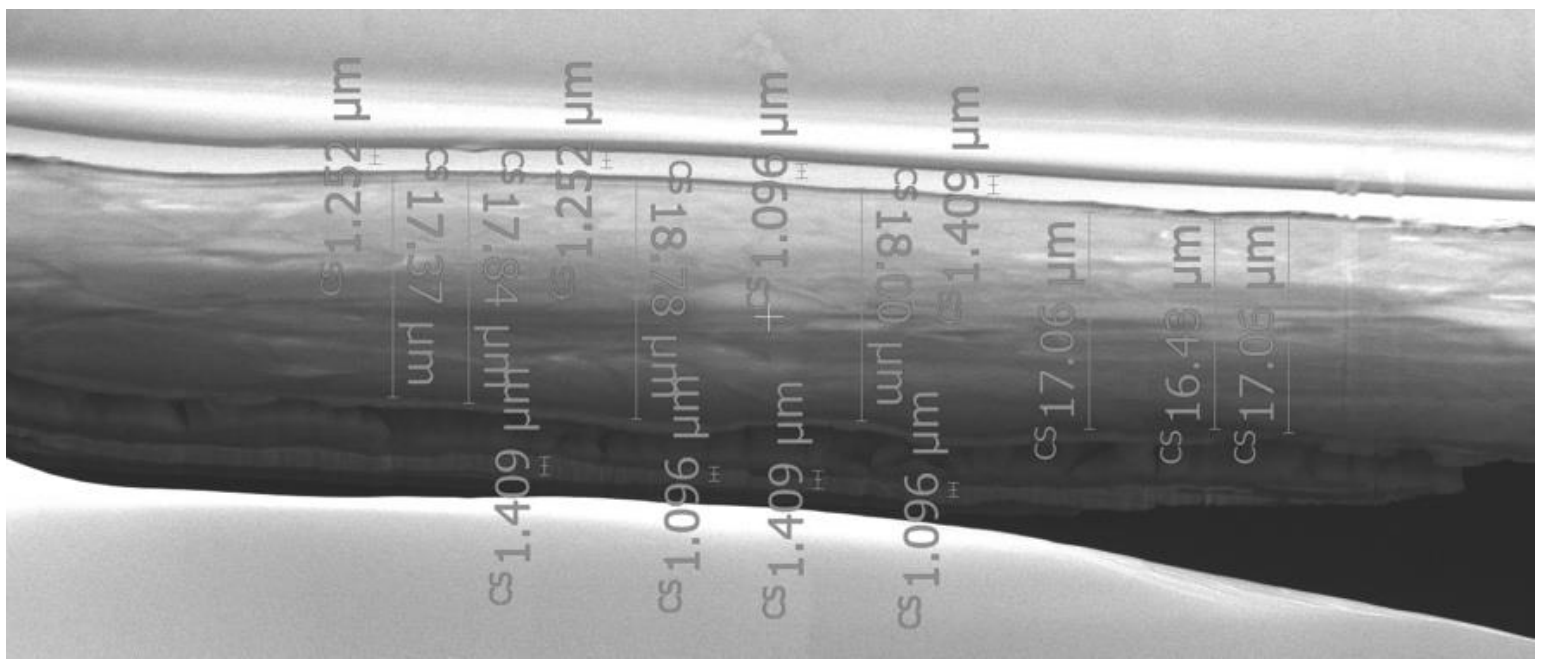

(SEM image of the cross-section of the 177 wt.% H_2O-bearing bridgmanite sample recovered from run #3)

Regarding the sample geometry when different electrodes are used, this is an important and insightful point. When using Pt electrodes, their relatively softer nature compared to bridgmanite allows them to deform preferentially, resulting in improved geometric control of the sample. In contrast, the harder BDD may locally indent into the sample under compression, leading to greater thickness heterogeneity. As a potential improvement, introducing a thin metal interlayer (e.g., Ir, Pt, or Au) between the sample and BDD could help maintain better geometry while preserving the advantages of BDD as both an electrode and laser absorber. We thank the reviewer for this valuable input.

7. It would be straightforward for comparison if authors can include their own EC measurement of dry bridgmanite from their previous study.

We appreciate the reviewer's suggestion. We are currently pursuing such measurements as part of ongoing work, and the results will be reported in a future study.

Minor Comments:

L126. 'high-temperature EC data extrapolated to room temperature' maybe easier for reader to change to 'room temperature EC data extrapolated from high-temperature data'.

We have modified it accordingly.

L152. Why exponent of Cw is 1/3? Maybe add references? L157. 'Previous studies'. Add references. L213. '1-2 orders of magnitude'. Add references.

We have added all the references accordingly in the revised manuscript.

We thank the reviewer again for their careful and valuable comments, which have significantly improved the manuscript.

Response to the comments by Reviewer #2 (reviewer comments in bold and our response in blue):

I appreciate the work presented in this study and recognize the technical challenges involved in developing such an experimental protocol. However, there are several critical components missing from the manuscript that need to be addressed before it can be considered further.

We thank the reviewer for recognizing the technical challenges of the presented measurements and the significance of our study. Below, we address the concerns raised regarding the robustness of our results.

The clearly defined tails observed in the impedance spectra indicate the formation of a secondary phase (s) during the course of the experiments. It is important to note that impedance spectra are highly sensitive to chemical and physical changes occurring within the sample; therefore, even minute deviations in spectral shape should not be left unaddressed.

We thank the reviewer for this important comment. We do not interpret the low-frequency tails as evidence for the formation of a secondary phase; instead, they are consistent with electrode polarization under blocking-electrode conditions.

In our experiments, impedance is measured using blocking electrodes (BDD or Ir). In such systems, the expected impedance response consists of (i) a semicircular arc corresponding to the intrinsic bulk response of the sample and (ii) a low-frequency contribution arising from electrode polarization associated with a double-layer formation. This behavior is well established for ionic conductors with blocking electrodes and can be described by a constant-phase element in series with the bulk resistance, resulting in a semicircle followed by a low-frequency linear feature.

In the original manuscript, room-temperature impedance measurements (run #2) showed a semicircular response together with an emerging low-frequency feature, as shown in the figure below; however, the conductivity was insufficient to fully resolve this component within the accessible frequency range.

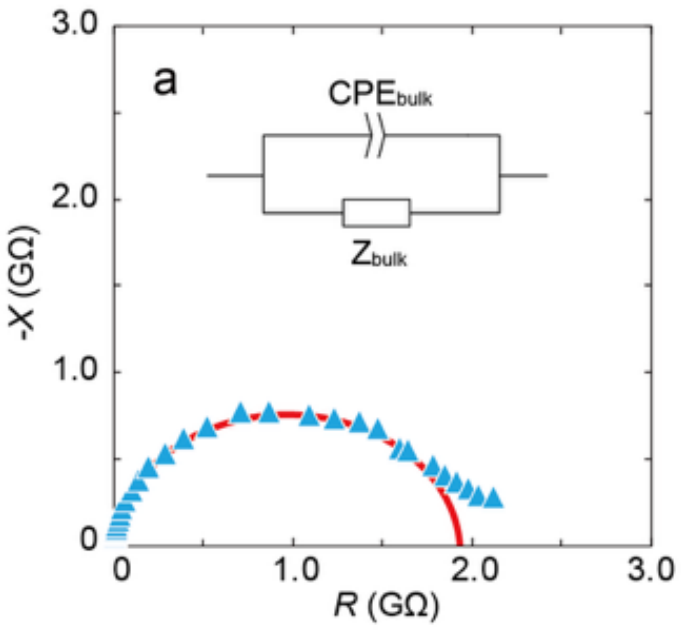

(Room temperature impedance data for 293 wt. ppm H_2O bearing bridgmanite in run #2)

To further evaluate the origin of this feature, we performed additional impedance measurements at high *P*–*T* (run #3 in the revised manuscript). These data clearly exhibit a semicircle followed by a well-defined low-frequency straight-line feature.

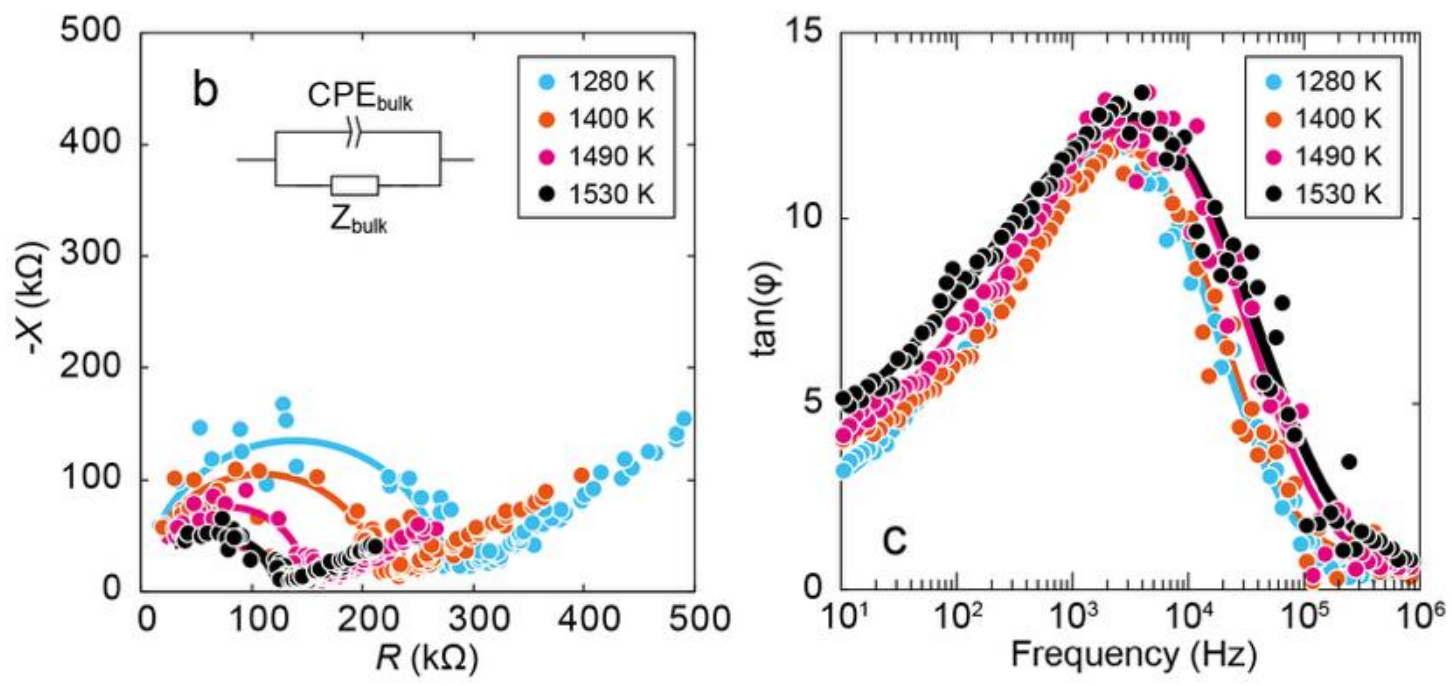

(High *P*–*T* impedance data for 177 wt. ppm H_2O bearing bridgmanite in run #3 in the revised manuscript)

Note that this low-frequency response is characteristic of electrode polarization rather than diffusion-limited (Warburg-type) behavior, which would produce an approximately 45° slope. The absence of additional semicircular features also argues against the presence of secondary phases or grain boundary contributions. Furthermore, no low-frequency "hook," often associated with dehydration, fluid migration, or excessive input voltage, is observed.

Importantly, such electrode polarization is a hallmark of *ionic* conduction in blocking-electrode configurations. In contrast, charge transport dominated by *electronic* carriers would produce only a semicircular response without a pronounced low-frequency tail. The diffusivity independently derived from this low-frequency feature is consistent with that obtained from electrical conductivity (Figure 4), further supporting its interpretation as arising from proton migration. We further consulted with an electrochemistry expert

(Prof. Rafał Jurczakowski, Warsaw University), who confirmed that the observed low-frequency response is most consistently explained by a double-layer formation associated with proton migration at the electrode–sample interface.

Taken together, these observations demonstrate that the low-frequency feature in our impedance data originates from interfacial polarization.

Furthermore, the evolution of the spectra with increasing temperature provides crucial information about the processes taking place in the sample, e.g. dehydration, melting, diffusion, reactions etc...

The clear deviation from ideal semicircular behavior suggests the formation of additional phase(s), possibly related to fluid or melt generation, or to chemical segregation. The authors should discuss this point in greater depth, particularly in relation to the temperature evolution of the impedance spectra.

Following the reviewer's suggestion, we performed additional impedance measurements on hydrous bridgmanite at high *P*–*T* conditions. As described earlier, the resulting spectra exhibit a semicircular arc, corresponding to the intrinsic bulk response of the sample, together with a low-frequency linear component arising from electrode polarization associated with a double-layer formation due to proton migration at the electrode–sample interface.

We agree that, in general, the formation of additional phases (e.g., melt or fluid) may influence impedance spectra. However, such processes typically introduce additional semicircular features, often associated with fluid migration in grain boundary and/or interfacial transport pathways. In contrast, the high *P*–*T* impedance data obtained in this study do not show additional semicircles but instead display a non-45° straight-line feature at low frequencies. This response is consistent with electrode polarization and cannot be explained by fluid or melt formation, which would be expected to produce a distinct semicircular contribution. It is also inconsistent with Faradaic electrode reactions involving protons, which would typically produce either an inductive loop or a Warburg-type response with an approximately 45° slope.

While the room-temperature impedance data in the original manuscript did not fully resolve the low-frequency feature, the newly obtained high *P*–*T* data clearly distinguish its nature. Taken together, these observations indicate that the deviation from ideal semicircular behavior arises from interfacial polarization rather than the formation of secondary phases. The high *P*–*T* impedance data and corresponding interpretation have been added to the revised manuscript.

In addition, the frequency range should be extended to lower frequencies (0.1 or 0.001 Hz) to better capture and interpret the processes occurring in this regime. Such misinterpretations might otherwise lead to unrealistic conductivity estimates and compromise the quality of the discussion.

We agree that extending the frequency range to lower frequencies for the room-temperature data would better capture processes occurring in this regime. As clarified by the additional high *P–T* impedance measurements, the low-frequency response is consistent with proton migration at the blocking electrode interface.

However, acquiring impedance data at such low frequencies under high *P–T* conditions is practically challenging. In LHDAC experiments, it is generally necessary to minimize heating duration to avoid unintended chemical reactions or segregation. Moreover, because we used a bridge-type impedance analyzer, the acquisition time for a single data point scales as >1/f; for example, measurements at 0.1 Hz and 0.001 Hz require at least ~10 s and ~100 s per data point, respectively. Since impedance spectra require frequency sweeps with multiple measurements for adequate signal quality, extending to such low frequencies would substantially increase total acquisition time. This makes such measurements difficult to implement under high *P–T* conditions.

We also explored the use of a frequency response analyzer, which enables more efficient frequency sweeps, for high *P–T* measurements. However, under high-temperature DAC conditions, such measurements are more sensitive to noise and open/short compensations. We found that the resulting impedance data were not sufficiently reliable for quantitative analysis. For this reason, we adopted a bridge-type impedance analyzer (HIOKI 3532-80) for the high *P–T* measurements, which, although slower, provides more robust and reproducible results under the present experimental conditions.

Moreover, the axial direction may not be the most appropriate orientation to capture chemical segregation that can occur in DAC experiments involving laser heating. The chemical analysis of the experimental run products should also be performed on a plane perpendicular to the axial direction to assess potential effects of temperature gradients.

We agree that chemical segregation in laser-heated DAC experiments, particularly along thermal gradients, should be carefully evaluated. In our study, we did not observe any evidence of chemical segregation, including in the radial direction, as confirmed by the analysis of the recovered samples (Supplementary Figure 7 in the revised manuscript). If present, segregation is typically most pronounced near the rim of the laser-heated spot due to strong radial temperature gradients. However, within the resolution of our post-

experimental analyses, the sample appears chemically homogeneous, and no systematic heterogeneity was detected. These observations suggest that any potential segregation effects are minimal under our experimental conditions. We note that such effects may become more significant at substantially higher temperatures (e.g., >3000 K), where enhanced diffusion could promote chemical segregation.

Finally, the distance between the two electrodes appears to vary significantly. The authors should clarify how such deformations were accounted for during data analysis and conductivity calculations.

We thank the reviewer for this important comment, which was also raised by another reviewer. We consider that the use of hard BDD electrodes may lead to local indentation into the sample during compression, as BDD is mechanically stronger than the bridgmanite sample. This could result in increased thickness heterogeneity compared to our previous studies.

To account for this effect, the sample thickness used in conductivity calculations was determined as the average thickness within the laser spot region. The associated uncertainty was estimated from the standard deviation of these measurements and was at most 6.5%. This uncertainty was propagated into the conductivity values. Therefore, the effect of thickness heterogeneity is quantitatively incorporated in our reported error estimates.

While the theoretical predictions provide a valuable and interesting dimension to the discussion, it is important that they do not substitute for a clear and detailed description of the data acquisition process and experimental protocols, as these aspects are essential to ensure the accuracy and reliability of the results.

We appreciate the reviewer for this comment and for the opportunity to clarify the basis of our analysis. We note that the present study is based on experimental measurements, and the conclusions are derived directly from the observed data. The data acquisition procedures and experimental protocols were clearly described in the original manuscript. The consistency of the results is supported by several independent observations. Measurements on samples with different water contents show a systematic, approximately linear dependence of conductivity on water content. Impedance spectroscopy is also consistent with proton-dominated conduction, based on the characteristic low-frequency response associated with electrode polarization under blocking-electrode conditions (i.e., confirming that charge carriers are not electrons). In addition, diffusivities independently derived from electrical conductivity and from

analysis of the low-frequency impedance feature are in agreement for both room-temperature and high *P*–*T* data. Together, these observations support the reliability of the measurements and their interpretation.

We thank the reviewer again for their careful and valuable comments, which have significantly improved the manuscript.

Response to the comments by Reviewer #3 (reviewer comments in bold and our response in blue):

The authors measure the electrical conductivity (EC) of hydrous bridgmanite and use these results to infer water content in the lower mantle. This is an important topic, the high P–T EC measurements appear robust, and the manuscript is generally well written. But I have concerns on starting sample, water concentration, and how the inclusion affects EC results.

We appreciate the reviewer's positive assessment of our manuscript. We address the concerns regarding the starting material, water concentration, and potential effects of inclusions below.

First, a few previous relevant studies need to be included in the paper:
Amulele et al. 2021, JGR Solid Earth, https://doi.org/10.1029/2021JB022222
Zhang et al. 2025, JGR Solid Earth, https://doi.org/10.1029/2024JB030403
Lu et al. 2025, Science, DOI: 10.1126/science.adx5883

Thank you for your input. We have added these papers accordingly in the revised manuscript.

The starting bridgmanite sample shows clear contamination from brucite inclusions (Supplementary Fig. 4; Lines 409-411). The authors report a lower water content compared to Ref. 33, but it is unclear whether this discrepancy reflects intrinsic solubility differences or results from brucite inclusions or precipitates formed during quenching, as discussed in Refs. 33, 38, and Amulele et al. (2021). If such inclusions formed during quenching, then in experiments heated to high P-T conditions, water potentially released from brucite would further hydrate bridgmanite. Moreover, recent results by Lu et al. (2025) show that water solubility in bridgmanite increases with temperature, suggesting that progressive hydration during heating is likely. Under these conditions, a sharp jump in conductivity, as proposed in Lines 420-422, may not occur.

We appreciate the reviewer's insightful comment. We agree that, in principle, water released from brucite during heating could be incorporated into bridgmanite, potentially modifying its water content, especially in light of recent findings that water solubility in bridgmanite increases with temperature (Lu et al., 2025). However, we consider that this effect, if present, is limited under our experimental conditions. The measured electrical conductivity shows a linear dependence on temperature in Arrhenius space across the

entire temperature range, without any abrupt or progressive deviations that would indicate significant changes in water content during heating. In addition, the relatively high pressures of our experiments favor increased water solubility in bridgmanite, reducing the likelihood of transient fluid accumulation along grain boundaries.

To clarify this point, we have added a discussion in the revised manuscript on page 16, lines 412–419, as follows:

"[…] brucite may decompose into MgO+H_2O under the P-T conditions of this study[80], which could cause an irreversible jump in conductivity due to highly conductive H_2O fluid in grain boundaries[81]. Nonetheless, no such conductivity jump was observed in this study (see Results for details). Recent studies also indicate that the water solubility of bridgmanite increases with temperature[71], raising the possibility that water released from brucite could be incorporated into bridgmanite, gradually modifying its water content and, in turn, its EC. However, the measured conductivity shows a linear dependence on temperature, suggesting that such rehydration effects did not significantly influence the results."

The authors state that inclusions "do not affect EC as long as they are not interconnected" (Lines 417-418). However, the inclusions appear to be in direct contact with the bridgmanite matrix. Given that brucite has a lower electrical conductivity than bridgmanite (Line 419), such inclusions could bias the measurements toward lower EC values.

We agree that non-conductive and isolated inclusions can lead to an underestimation of the bulk conductivity. However, the magnitude of this effect depends on the volume fraction of the inclusions. Their influence on the effective conductivity can be approximated using effective medium bounds (Hashin–Shtrikman bounds, isolated model). Even in an extreme case of ~10 vol.% brucite, the resulting reduction in conductivity would be on the order of ~10%. In our samples, brucite was not detected in the XRD patterns, indicating that, if present, its abundance is at most a few vol.% given the detection limit for minor phases. Therefore, the impact of brucite inclusions on the measured conductivity is expected to be minor, at most ~ a few %.

We have added a corresponding discussion in the revised manuscript on page 16, lines 405–412 as follows.

"The inclusion of a minor phase does not significantly affect EC measurements as long as it is not interconnected in a sample; the EC of brucite at high P-T conditions has been reported to be lower than that of bridgmanite[78], and thus isolated brucite inclusions may slightly underestimate the bulk conductivity. Brucite was not detected in the XRD patterns,

indicating that, if present, its abundance is at most a few vol.%. Based on effective medium bounds for isolated inclusions[79], such a small volume fraction would result in an underestimation of up to only a few percent, suggesting a minor effect on the measured conductivity."

The authors use Paterson's calibration (Equation 6) to estimate water content. However, this calibration is known to be inappropriate for many silicate minerals, including bridgmanite, as discussed in Zhang et al. (2025). Application of this calibration may therefore introduce systematic errors in the estimated water concentrations, which directly affects the interpretation of the water effects on EC.

We thank the reviewer for this important comment. We agree that the applicability of the Paterson calibration to bridgmanite is limited. Zhang et al. (2025) has shown that the absorption coefficient of bridgmanite deviates from the Paterson calibration, such that the use of this calibration may lead to a systematic underestimation of water content.

In the present study, we use the Paterson calibration as a first-order estimate due to the absence of independent constraints on water content (e.g., SIMS-based measurements). Therefore, the absolute values of water content should be regarded with caution. Since that affects the maximum water content of the lower mantle we estimated here, we have added a corresponding text about water concentration in the revised manuscript (page 16, lines 395–399).

"These values are lower than those reported for hydrous (Fe,Al)-bearing bridgmanite[33], likely due to differences in iron and aluminum content (Supplementary Fig. 5), as well as a potential systematic underestimation of water content by the Paterson calibration, as recent work suggests that the absorption coefficient of bridgmanite deviates from the Paterson calibration[50]."

Please add error bars in Figs. 2-4. Also, what is the error bar for the threshold of 40 ppm H2O (line 38 and elsewhere)?

Error bars were included in Figs. 2–3 and were shown only for the highest *P–T* datum in each run for clarity; however, this was not clearly stated in the original figure captions. We have now revised the captions of Figs. 2–3 to explicitly indicate this.

For Fig. 4, we were only illustrating error bars for electrical conductivity that were all hidden behind the symbols. In the revised manuscript, we made each symbols smaller so that we can see the error bars, and also added discription how we estimated the uncertainties in the figure caption. Regarding the threshold value of 40 ppm H_2O, we have

included the associated uncertainty of 40 ± 11 ppm H_2O based on electrical conductivity uncertainties.

We are grateful to the reviewer for their careful and insightful comments, which have helped improve the manuscript.

Response to the comments by Reviewer #1 (reviewer comments in bold and our response in blue):

The authors conducted the additional impedeance measurement at high temperature and the results are consisent with the resistance measurement.
In additon, the authors have added a run with Ir electrode to prove the effect of potential breakage of BDD electrode on the results are small.
Therefore, I have no further comments on the manuscript.
We thank the reviewer for their positive evaluation of our revised manuscript and for acknowledging the additional high-temperature impedance measurements and the additional run using Ir electrodes. We are pleased that the reviewer has no further comments.

Response to the comments by Reviewer #4 (reviewer comments in bold and our response in blue):

This manuscript presents an important new conductivity dataset for hydrous Fe-Al-bearing bridgmanite at lower-mantle conditions. The experimental work is valuable, the conductivity measurements appear technically sound, and the manuscript is generally well written. Comparing with geophysical conductivity observations has the potential to provide important constraints on water storage in the Earth's lower mantle. Overall, the authors have addressed the previous reviewers' comments well and the manuscript is now in much better shape. However, I still have concerns regarding the inferred <40 ± 11 ppm H_2O constraint (Supplementary Fig. 3):

We thank the reviewer for their positive evaluation of our revised manuscript and for acknowledging the value of the new conductivity dataset, the technical soundness of the measurements, and the improvement of the manuscript after the previous round of revision. We also appreciate the reviewer's constructive concern regarding the robustness of the inferred <40 ± 11 ppm H_2O constraint. Our detailed responses to this concern are provided below.

This estimate appears to be based on extrapolation from only two hydrous compositions (177 and 293 ppm H_2O), which limits the ability to evaluate the conductivity–water relationship outside the measured range. More importantly, the manuscript itself suggests that conductivity may depend not only on water content but also on Fe-Al composition, as discussed in the comparison with Yoshino et al. If conductivity is sensitive to both water content and composition, it is unclear whether a conductivity–water relationship derived from a single bridgmanite composition can be uniquely applied to infer lower-mantle water contents.

This is a very good point, and we thank the reviewer for raising this important issue. In the previous version of the manuscript, we did not explicitly state that the inferred water-content estimate does not account for possible compositional effects of Fe and Al on the proton conductivity of bridgmanite. We agree that the conductivity-water-content relationship derived from a single bridgmanite composition in this study cannot be uniquely applied to all lower-mantle bridgmanite compositions without considering this assumption.

We now clarify that the inferred water-content threshold should be regarded as a first-order estimate applicable to lower-mantle bridgmanite with Fe-Al contents comparable to those of our experimental samples. We also note that the composition of our samples

is comparable to that expected for bridgmanite in a pyrolitic lower-mantle assemblage. Therefore, although the inferred water-content threshold should not be interpreted as a composition-independent universal constraint, we consider it to provide a reasonable first-order estimate for pyrolitic lower-mantle bridgmanite. We have revised the relevant text in the manuscript to clarify these assumptions and limitations on page 12, lines 295–301 in the revised manuscript, as follows.

"This estimate assumes that the Fe-Al content of lower-mantle bridgmanite is comparable to that of our experimental samples and to that expected for bridgmanite in a pyrolitic lower-mantle assemblage[68,69]. As discussed above, the comparison with Yoshino et al. suggests that proton conductivity in bridgmanite may also be sensitive to Fe-Al composition. If lower-mantle bridgmanite has substantially different Fe-Al contents or Fe valence states[70], the inferred water-content threshold may change accordingly."

In addition, the manuscript states that below ~40 ppm H2O, small-polaron conductivity exceeds proton conductivity. However, the ~40 ppm threshold appears to be defined by the intersection of an extrapolated hydrous bridgmanite conductivity trend and the conductivity of pyrolite reported by Ohta et al. Because pyrolite conductivity represents a bulk multiphase assemblage rather than dry bridgmanite itself, it is unclear whether this intersection uniquely defines the transition from proton-dominated to small-polaron-dominated conduction in bridgmanite or can be directly translated into a water-content threshold for lower-mantle bridgmanite.

Taken together, it is unclear how robustly a quantitative constraint of <40 ± 11 ppm H_2O can be inferred from the available data. At a minimum, the assumptions underlying this estimate should be discussed more explicitly.

We thank the reviewer for this very important point. We agree that the conductivity of pyrolite reported by Ohta et al. represents a bulk multiphase assemblage and therefore does not uniquely define the transition between proton-dominated and small-polaron-dominated conduction in bridgmanite itself. In the previous version of the manuscript, we used this pyrolite conductivity as a reference because bridgmanite is expected to largely control the bulk conductivity of a pyrolitic aggregate, as suggested by Yoshino et al. (2008; ref. 14 in the main manuscript). However, we agree that this approach does not provide a phase-specific dry bridgmanite reference. We have therefore revised the manuscript to clarify this limitation and no longer treat the previously inferred <40 ± 11 wt. ppm H_2O value as a uniquely defined quantitative constraint for bridgmanite.

In the revised Supplementary Fig. 3, we now provide an additional comparison using the dry (Fe,Al)-bearing bridgmanite conductivity reported by Xu et al. (1998). Although the Al content of the bridgmanite studied by Xu et al. is lower than that expected for pyrolitic lower-mantle bridgmanite, this dataset provides a useful phase-specific reference for small-polaron conductivity in dry (Fe,Al)-bearing bridgmanite. Using this bridgmanite reference, the crossover between the extrapolated proton conductivity of hydrous bridgmanite and the small-polaron conductivity of dry (Fe,Al)-bearing bridgmanite occurs at 67 ± 18 wt. ppm H_2O. For comparison, the corresponding crossover using the pyrolite conductivity of Ohta et al. occurs at 40 ± 11 wt. ppm H_2O.
Therefore, rather than treating <40 ± 11 wt. ppm H_2O as a uniquely defined composition-independent constraint, we now describe the crossover as occurring at approximately ~40–70 wt. ppm H_2O.
We have revised the relevant text in the main manuscript on pages 11–12, lines 289–295, as follows:
"At these depths, we found that, below ~40–70 wt. ppm H_2O, small-polaron conductivity of dry Fe,Al-bearing bridgmanite[19] or dry pyrolite[23] exceeds proton conductivity estimated from hydrous bridgmanite (Supplementary Fig. 3). This crossover indicates that, below this water content, the bulk conductivity of bridgmanite is expected to be dominated by small-polaron conduction rather than proton conduction, making it difficult to resolve lower water contents from conductivity alone. We therefore conclude that bridgmanite in these depth ranges contains <40–70 wt. ppm H_2O."

We have also revised Supplementary Fig. 3 and its caption to clarify how the ~40–70 wt. ppm H_2O range was obtained:

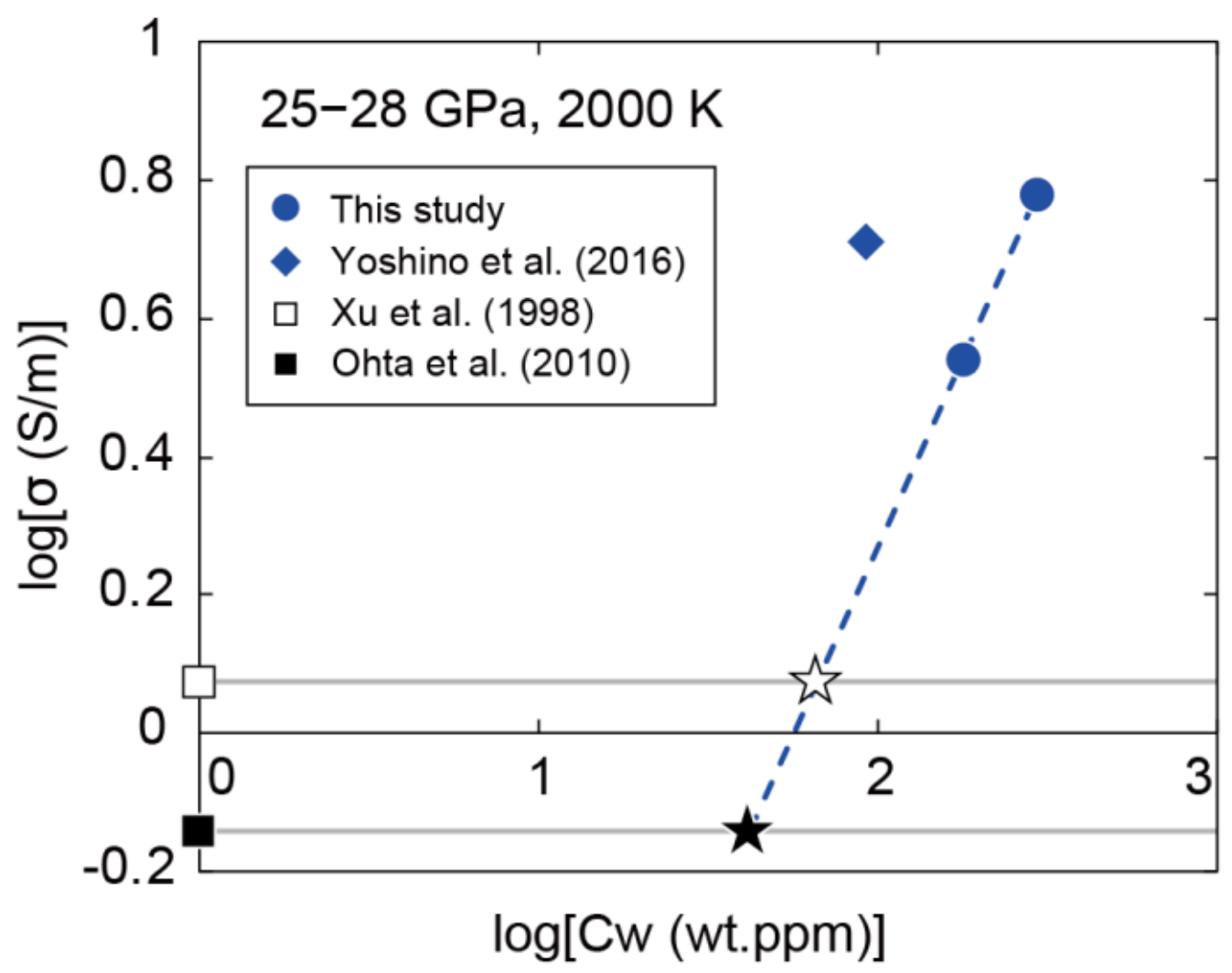

Supplementary Fig. 3 | Electrical conductivity of bridgmanite as a function of water content at 25–28 GPa, 2000 K. *Blue circles show the electrical conductivity of bridgmanite containing 293 and 177 wt. ppm H_2O-bearing bridgmanite (this study), and the blue diamond show 92 wt. ppm H_2O-bearing bridgmanite[13]. Black open and filled squares represent the electrical conductivity of dry (Fe,Al)-bearing bridgmanite[4] and dry pyrolite[2], respectively. Black open and filled stars indicate the estimated water contents at which proton conduction in hydrous bridgmanite equals the small-polaron conductivity of dry (Fe,Al)-bearing bridgmanite[4] and dry pyrolite[2], respectively, based on linear extrapolation of our data. The crossover occurs at 67 ± 18 wt. ppm H_2O when using dry (Fe,Al)-bearing bridgmanite[4] as the reference and at 40 ± 11 wt. ppm H_2O when using dry pyrolite[2] as the reference. These nominal crossover values suggest that bridgmanite with less than ~40–70 wt. ppm H_2O will predominantly exhibit small-polaron conduction rather than proton conduction. Therefore, this range represents an approximate lower limit of water content that can be resolved by comparing laboratory electrical conductivity measurements with geophysical observations.*

In addition, we considered the very recent electrical conductivity data for a pyrolite aggregate reported by Trubowitz et al. (2026). Because that study did not report Arrhenius fitting parameters that would allow extrapolation to the pressure-temperature conditions used in Supplementary Fig. 3, we did not use those data in the crossover calculation. However, their pyrolite conductivity is broadly consistent with the pyrolite conductivity reported by Ohta et al. at comparable lower-mantle conditions. This suggests that the water-content estimate based on the Ohta et al. pyrolite reference is unlikely to change substantially if the new pyrolite aggregate data are considered. To incorporate this recent dataset, we have added the laboratory-based conductivity profile of Trubowitz et al. to Fig. 4 of the main manuscript, as follows.

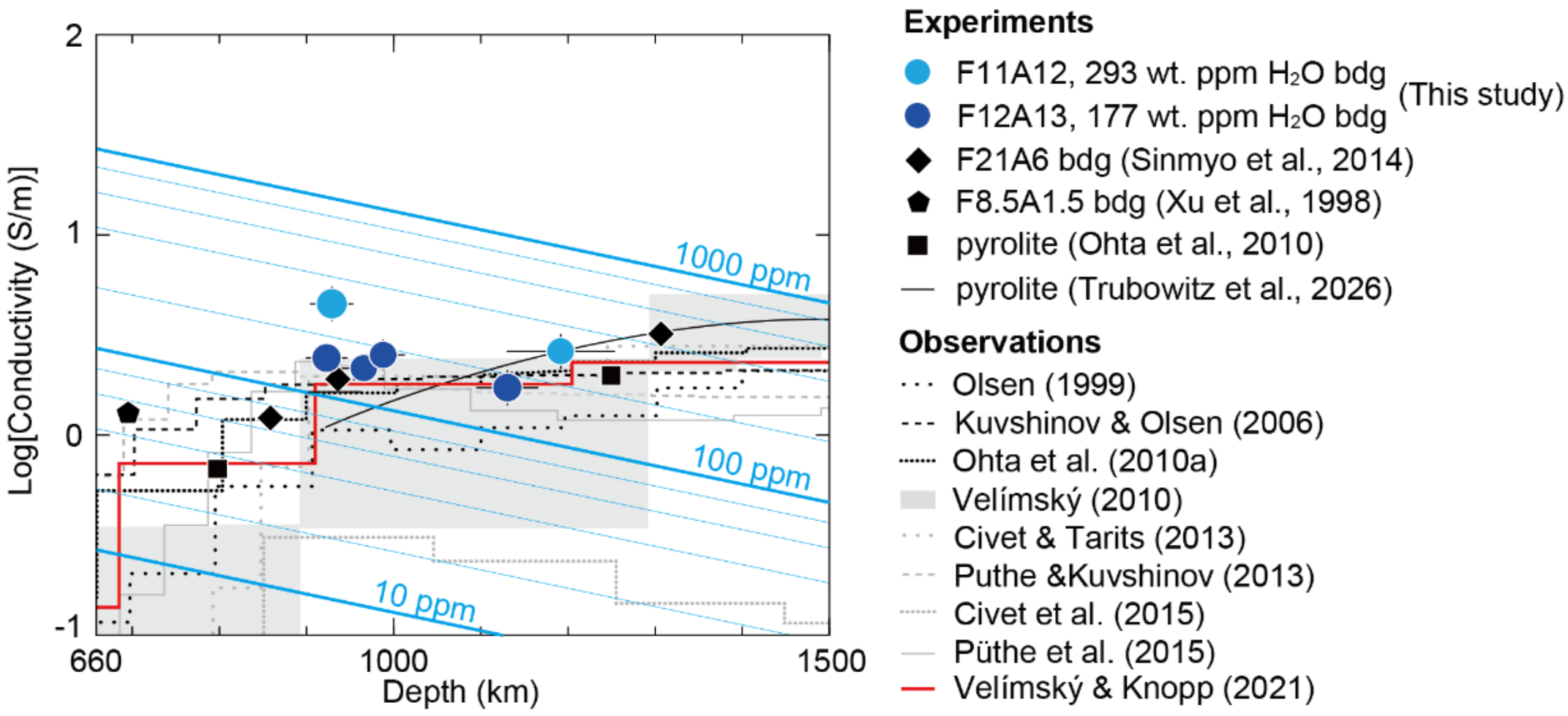

Fig. 4 | One-dimensional depth profiles of the electrical conductivity of hydrous bridgmanite in the Earth's lower mantle. *Light blue and dark blue symbols represent the electrical conductivity of 293 and 177 wt. ppm H_2O-bearing bridgmanite (bdg). Blue lines represent the estimated proton conductivity of bridgmanite at 10, 100 and 1000 wt. ppm H_2O, with additional minor lines at intermediate values evenly spaced on the logarithmic scale. Error bars on conductivity represent uncertainties propagated from experimental measurements. Depth uncertainties were derived from pressure uncertainty. Black square, diamond, and pentagon represent the previous laboratory measurements of pyrolite[23], (Fe,Al)-bearing bridgmanite[28], and Fe 8.5, Al 1.5 mol% bridgmanite[19], respectively. Black solid curve represents the previous laboratory-based conductivity profile for pyrolite[104]. Black dotted, broken, and dense dotted lines, and gray band, dotted, broken, dense dotted, and solid lines represent the profiles obtained by the Occam inversion from the observed C-response data from Olsen[60], Kuvshinov & Olsen[59], Ohta et al.[23], Velímský[63], Civet & Tartis[58], Püthe & Kuvshinov[62], Civet et al.[57], and Püthe et al.[61], respectively. Red line represents the most recent observation data (CUP20-OSC5 model) from Velímský & Knopp[64].*

We thank the reviewer again for their careful and valuable comments, which have significantly improved the manuscript.
